# Gravitational forces and matrix stiffness modulate the invasiveness of breast cancer cells in bioprinted spheroids

**DOI:** 10.1016/j.mtbio.2025.101640

**Published:** 2025-03-04

**Authors:** Louise Breideband, Kaja Nicole Wächtershäuser, Ryan Sarkar, Melosha Puspathasan, Ernst H.K. Stelzer, Francesco Pampaloni

**Affiliations:** Biological Sciences (IZN), Buchman Institute for Molecular Life Sciences (BMLS), Goethe-Universität Frankfurt am Main, DE-Frankfurt am Main, Germany

**Keywords:** Bioprinting, Breast cancer, Microgravity, Extracellular matrix, Tumor microenvironment, Tumor Invasiveness

## Abstract

The progression of breast cancer is influenced by the stiffness of the extracellular matrix (ECM), which becomes stiffer as cancer advances due to increased collagen IV and laminin secretion by cancer-associated fibroblasts. Intriguingly, breast cancer cells cultivated in two-dimensions exhibit a less aggressive behavior when exposed to weightlessness, or microgravity conditions. This study aims to elucidate the interplay between matrix stiffness and microgravity on breast cancer progression. For this purpose, three-dimensional spheroids of breast cancer cell lines (MCF-7 and MDA-MB-231) were formed. These spheroids were subsequently bioprinted in hydrogels of varying stiffness, obtained by the mixing of gelatin methacrylate and poly(ethylene) glycol diacrylate mixed at different ratios. The constructs were printed with a custom stereolithography (SLA) bioprinter converted from a low-cost, commercially available 3D printer. These bioprinted structures, encapsulating breast cancer spheroids, were then placed in a clinostat (microgravity simulation device) for a duration of seven days. Comparative analyses were conducted between objects cultured under microgravity and standard earth gravity conditions. Protein expression was characterized through fluorescent microscopy, while gene expression of MCF-7 constructs was analyzed via RNA sequencing. Remarkably, the influence of a stiffer ECM on the protein and gene expression levels of breast cancer cells could be modulated and sometimes even reversed in microgravity conditions. The study's findings hold implications for refining therapeutic strategies for advanced breast cancer stages - an array of genes involved in reversing aggressive or even metastatic behavior might lead to the discovery of new compounds that could be used in a clinical setting.

## Graphical abstract

**Figure:**
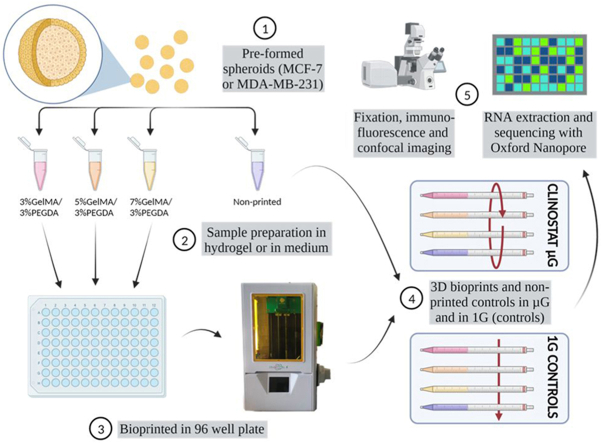

## 1 Introduction

The tumor microenvironment, composed mainly of immune cells, blood vessels, stromal cells and extracellular matrix, is known to constantly interact with and influence the cancer cells [1,2]. Cancer cells have primarily been studied in two dimensional (2D) conditions for *in vitro* studies. The addition of a second or third cell type, or the culture in three-dimensions (3D) in a matrix have only gained popularity in the last two decades [3] and are still not prevalent in oncology studies, drug testing assays or regulated preclinical trials [4]. With regulatory bodies like the Food and Drug Administration in the USA promoting non-animal models, interest in 3D cultures that mirror human physiology is increasing [5]. 3D cultures offer advantages over 2D by mimicking tissue-like interactions [[6], [7], [8]] but they face challenges in controlling mechanical properties such as stiffness and porosity [9,10]. The use of 3D bioprinting helps overcome many of these issues. Indeed, bioprinting enables the use of synthetic bioinks with specific mechanical properties. Bioinks, the material used in bioprinters, are composed of cells embedded in a matrix recapitulating the extracellular matrix (ECM). With modalities depending on the chemical properties of the bioinks and the type of bioprinting technology used, a spatially controlled crosslinking of the matrix is achieved, encapsulating the cells in place. The bioinks chemical composition can be tailored to control the mechanical properties of the matrix, allowing replication of the viscoelasticity of various tissues, from soft (e.g. brain tissue [[11], [12], [13]]) to hard (e.g. bone [[14], [15], [16]]). Moreover, state-of-the-art bioprinting techniques can generate gradients of mechanical properties within a single construct [[17], [18], [19]], which is particularly useful for studying transitions in tissue properties, which is highly relevant in cancer biology. By using computer-aided design (CAD), a 3D bioprinter can print a vast array of shapes in a highly reproducible manner, replicating the microarchitecture of specific organs or tissues. A CAD design can be easily modified within minutes – this level of flexibility is not attainable with other tissue engineering methods, for example using molds. This facilitates a fast-pace iterative approach leading to more accurate models for disease study and drug testing [20]. Finally, 3D bioprinting allows the production of a high number of replicates, adding more statistical significance.

Breast cancer (BC) is the most prevalent cancer and one of the leading causes of cancer-related death in women [21]. BC are very heterogenous and divided into subtypes based on their localization (basal or luminal) and the presence of specific receptors: estrogen receptor (ER), progesterone receptor (PR) and human epithelial receptor 2 (HER2). The classification therefore includes basal and luminal A and B types, HER2 positive and triple negative (ER-PR-HER2-), the latter being the most aggressive and metastatic [22,23]. The subtypes can exhibit similar or overlapping hormonal or molecular expression but lead to different clinical outcomes, although these are mainly determined by the stage of the tumor [24]. The correlation of BC progression and extracellular matrix (ECM) mechanics has previously been established. Similar to events taking place during embryonic development, the composition and stiffness of the ECM determines cellular behavior during cancer progression by recruiting cancer-associated fibroblasts (CAF) to deposit and remodel ECM proteins [25]. Through this process, the ECM becomes stiffer and richer in collagen fibers which in turn influences the BC cell fate [26,27]. Once again, these interactions are mostly absent when using 2D cell culture. To model these *in vivo* situations appropriately *in vitro*, the use of bioprinter combined with varied composition of bioinks would be an optimal approach.

One of the bioprinting techniques that can provide the most flexibility, upscaling possibilities and highest resolution is digital light processing (DLP) stereolithography (SLA). SLA works in a layer-by-layer fashion in which the pattern for each layer is projected onto a thin volume of light-sensitive bioink in conjunction with a platform moving upwards [28]. One method to control the stiffness of the bioprinted object is to change the concentration of photosensitive polymers within the bioink – the higher the concentration, the stiffer the scaffold [15,29].

While the influence of ECM's mechanical properties on the progression of breast cancer and other tumors is now widely recognized, recent studies show that gravity likewise influence the phenotype of tumor cells [30,31]. While achieving real microgravity is only possible in Earth orbit or deep space and requires substantial resources, simulated microgravity can be achieved under conventional laboratory settings by zeroing the gravity vector using motion [32]. Several approaches to simulated microgravity are available. A 3D random positioning machine (RPM) rotates the sample randomly and at two different speeds around two axes, time-averaging gravity to zero [33,34]. A 2D clinostat rotates the sample around one axis at a constant speed realizing a state of free fall while preventing rotational forces [35,36]. The works cited in references [[22], [23], [24], [25]] report that after removing the Earth gravity vector by exposing adherent BC cells to simulated microgravity, groups of cells detached from the substrate to form 3D spheroids. The spheroids displayed a vast cytoskeletal remodeling and changes in cell-cell adhesion. Interestingly, the onset of cancer metastasis involves a similar reorganization of cell-cell and cell-ECM adhesions leading to the detachment of a group of cells from the tissue, a process known as epithelial to mesenchymal transition (EMT) [41]. Research on colorectal cancer cells (DLD-1) and leukemic cells (MOLT-4, HL-60) using a rotating cell culture system [42] equipped with high aspect-ratio vessels (HARV) showed altered cancer cell proliferation by dysregulation of oncogenes and cancer progression markers, particularly in the colorectal cancer cell line. Strikingly, the DLD-1 cells upregulated mir-22 microRNA under microgravity. Mir-22 functions as a tumor suppressor by targeting Myc Binding Protein (MYCBP) [43,44]. An increased autophagic flux and upregulation of stemness markers was detected in HCT116 cells (colon cancer) spheroids exposed to simulated microgravity in an RCCS. These changes were not observed in HCT116 spheroids cultured at 1g. A drastic rearrangement of the cytoskeleton is a common observation in cells cultured under microgravity. Particularly relevant for cancer biology are the changes occurring in the intermediate filaments. MDA-MB-231 human breast cancer cells were grown in a Random Positioning Machine (RPM). After 72 h of exposition to simulated microgravity, part of the cells displayed a rounded morphology and detached from the tissue culture substrate producing floating aggregates. The rounded morphology was accompanied by profound changes in cytoskeletal architecture, with disruption of actin microfilaments and microtubules. Interestingly, the vimentin network was also lost, and the appearance of dense perinuclear vimentin aggregates was observed. As vimentin is a marker of highly metastatic potential in breast cancer, the disruption of the vimentin network signalizes the development less malignant phenotype under microgravity conditions [45]. Overall, the body of published data suggest a less aggressive phenotype in microgravity environments for both solid and hematologic tumors. Thus, microgravity represents a unique platform to study tumor cells by providing an environment where the mechanical stresses exerted by Earth's gravity on tissues are eliminated. In other words, microgravity allows to isolate processes like cell adhesion and migration from the gravitational “noise”. The insights gained from microgravity assays can greatly contribute to our understanding of cancer progression and lead to the development of new therapeutic approaches [[32], [33], [34], [35], [36]]. The simulated microgravity experiments conducted so far prevalently rely on 2D cultures of cancer cells and free-floating spheroids. To our knowledge no investigations have been performed to date on 3D cell cultures, in which the cells are embedded in extracellular matrix with a well-defined bioprinted geometry [[37], [38], [39], [40], [41]].

In this study, BC 3D cultures were encapsulated in hydrogels with varying degrees of ECM stiffness using 3D bioprinting. As a proof of principle to test the bioprinting pipeline and the shape reproducibility, a cylindrical structure was designed and bioprinted. Next, the constructs were exposed to simulated microgravity (0g) for seven days. The cells were prepared as compact spheroids, which better mimic the architecture of tumors, including the presence of a necrotic core [46,47]. A low-cost, custom 3D bioprinter was used for the bioprinting process [29], while microgravity conditions were simulated using a 2D clinostat. Compared to their earth gravity (1g) counterparts and non-bioprinted controls, the cells cultured in 0g exhibited distinct responses to ECM stiffness, negating or even reversing the effect of the ECM. Our findings demonstrate that, in simulated microgravity (0g), the impact of the ECM matrix varied depending on the cell type. Additionally, the over- or underexpression of genes associated with aggressivity and metastasis in breast cancer was influenced not only by the gravitational condition (0g versus 1g) but also in combination with the presence or absence of the ECM. This underscores the limitations of traditional 2D models, which oversimplify these interactions and fail to capture the full complexity observed in 3D environments. Finally, state-of-the-art 3D models (spheroids) without any matrix (controls) showed little difference between 0g and 1g, indicating that 3D models without ECM were not sufficient to demonstrate significant differences.

These results suggest that incorporating simulated microgravity as an additional environmental factor in BC potentially allows to isolate the effect of matrix stiffness from other effects. Arrays of genes could be identified that were not previously flagged in BC publications. This could lead to the identification of novel therapeutic targets and provide deeper insights into tumor behavior.

## 2 Materials and methods

### 2.1 Cell culture

The breast cancer cells, MCF-7 and MDA-MB-231 (kindly gifted by PD Dr. Sjoerd J.L. van Wijk at the Institute for Experimental Pediatric Hematology and Oncology (EPHO), Goethe University Frankfurt) were obtained from ATCC and DSMZ respectively. The cells were cultured in RPMI medium (ThermoFisher Scientific) with 10 % qualified fetal bovine serum (FBS, Gibco ThermoFisher Scientific) and 1 % Penicillin/Streptomycin (PenStrep, ThermoFisher Scientific). The flasks used for cell cultures measured 75 cm^2^ (T75, Cellstar from Greiner Bio-One GmbH). The cells were kept at 37 °C, 5 % CO_2_ in a humidified incubator (Thermo Scientific Heracell 150i). All steps were performed in a sterile environment. The cells were passaged at approximately 90 % confluency and the medium changed every two to three days. The cells were used between passages 4 and 15. For passaging, the cells were washed with phosphate buffered saline (PBS pH 7.4, ThermoFisher Scientific) before being incubated for 5 min with Accutase (1 ml per T75 flask, Gibco ThermoFisher Scientific). The cell detachment was controlled then medium was added to the Accutase-cell mixture before centrifuging (300 rpm, 5 min). The supernatant was discarded, and the cell pellet was resuspended in fresh medium. A portion of the cell-medium mixture was transferred to a new flask to a ratio of 1:2 to 1:10, depending on when the experiment was due, with 10 ml fresh medium.

The cells were used to form spheroids for the experiments. Spheroids of 1000 cells were formed in a Sphericalplate 5D (Kugelmeiers Ltd.) two to four days before bioprinting. The spheroids were collected, the medium discarded and the spheroids were resuspended in one of the concentrations of hydrogel to a cell density of two million cells per milliliter (ml) before bioprinting (see 2.3). The control spheroids were not bioprinted but further processed for the microgravity experiments (see 2.5).

### 2.2 Hydrogel preparation

Gelatin methacrylate (GelMA), around 80 % bloom, (kindly gifted by Dr. Núria Torras, Institute for Bioengineering of Catalonia (IBEC), The Barcelona Institute of Science and Technology (BIST), Spain) was prepared using porcine skin type A. The preparation protocol has been previously described [19]. Poly(ethylene) glycol diacrylate (PEGDA), average Mn 4000, as well as the photoinitiator lithium-Phenyl-2,4,6-trimethylbenzoylphosphinat (LAP) and the photo-absorber tartrazine were purchased from Sigma Aldrich. SILAC (stable isotope labeling with amino acid) advanced DMEM/F-12 Flex Media, no phenol red and no additives (SILAC medium) was purchased from Gibco ThermoFisher Scientific.

GelMA and PEGDA with LAP were separately dissolved in SILAC medium at 65 °C under shaking for 2 h. SILAC medium helps support cells during bioprinting while also reducing errors linked to refractive index mismatch. GelMA and PEGDA/LAP were then mixed to a final concentration of GelMA of 3 %, 5 % and 7 % weight per volume (w/v%) with a constant concentration of PEGDA at 3 % w/v, LAP at 0.2 % w/v, and tartrazine at 0.003 % w/v and mixed for 1 h at 37 °C. In total, three conditions with three concentrations of GelMA were prepared. To simplify, the three concentrations will be described as 3 %, 5 % and 7 % (respectively 3 % w/v, 5 % w/v or 7 % w/v GelMA with 3 % w/v PEGDA, 0.2 % w/v LAP and 0.003 % w/v tartrazine).

### 2.3 Bioprinting

The bioprinting of spheroids was adapted from a previous protocol [29], using a modified Anycubic Photon S 3D printer (Anycubic Technology). The adaptations included a 96-well plate instead of an 8-well plate as a vessel for the bioink. To do so, a 96-well plate platform was designed (Autodesk Fusion 360 version 2.0.17721 x86_64), 3D printed (Bambu Lab X1-Carbon 3D printer) using PLA filaments and coated with aluminum (Weicon Aluminum-spray A-400). The design for the 96-well plate platform is available at the following link: https://www.thingiverse.com/thing:6969604 (Supplementary Fig. 1). An imaging 96 well plate (25 μm Fluocarbon Film Bottom, zell-kontakt GmbH) was used as a vat container. Cylinders (3.5 cm in diameter, 3 cm in height) containing branching channels were designed using Autodesk Fusion360 and were sliced using Anycubic Photon Workshop (Anycubic).

The slicing parameters for the bioprinting were defined as described in Table 1:

**Table 1:** Bioprinting parameters.

Layer thickness	150 μm
Normal exposure time	100 s
Off time	1 s
Bottom exposure time	150 s
Bottom layers	3
Z lift distance	3 mm
Z lift speed	9 mm/s
Z retract speed	9 mm/s

The bioprints were observed and imaged using a stereomicroscope (SteREO Discovery V8, Zeiss).

To bioprint, the spheroids were collected and resuspended in the light-sensitive hydrogel described previously in the three concentrations (3 %, 5 % and 7 %) to a final cell density of 4 million cells per milliliter. Six wells in the 96 well plate vat were filled per condition with 100 μl in each well. Two runs were consecutively bioprinted to utilize the total volume of bioink, producing up to 12 technical replicates per condition (some runs resulted in the absence of constructs on some platform, likely due to bioink drying or pipetting errors). After a bioprinting run, the objects were separated from the platform using a spatula and transferred to a 96-well plate (Falcon) containing PBS and 1:500 normocin (Invivogen) warmed to 37 °C, to wash the non-crosslinked hydrogel away and reduce the risk of contamination. After incubating for around 30 min, CO_2_-independent medium (ThermoFisher Scientific) with 1:500 normocin was added to the well.

### 2.4 Cell viability assay

The cell viability was first visually assessed using a live-dead assay. The dead cells were stained with propidium iodide (PI) at 1:100, the live cells with fluorescein diacetate (FDA) at 1:500 (Sigma Aldrich) and the nuclei with Hoechst 33342 at 1:500 (Thermo Fisher Scientific) in SILAC medium for 15 min at 37 °C (see Supplementary Table 2). The samples were processed directly after bioprinting (day 0) and again after seven days (day 7) incubation in CO_2_ independent medium in the same incubator containing the clinostat. They were imaged directly after staining on a confocal microscope AxioObserver LSM780 (Zeiss).

To be able to quantify the number of live cells after seven days in the clinostat, we used a CellTiter-Glo (CTG) assay (Promega). This assay allows the ATP produced by the living cells to be measured using luminescence. The cells were treated using the kit in CO_2_ independent medium and left at room temperature for 30 min, sheltered from light. Then, the luminescence was asserted using a plate reader (Infinite M200, Tecan). An integration time of 250 ms was selected. Blank wells, wells with medium only and wells with CTG and medium were used as controls. The samples were here again measured at day 0 and day 7.

### 2.5 Simulated microgravity

A 5 mL serological pipette (ThermoFisher Scientific) was repurposed as a vessel for use in a 2D rotational clinostat (Yuri GmbH, www.yurigravity.com). To prepare the pipettes, the ends were trimmed to achieve a final medium volume of approximately 6.5 mL; these modified pipettes are hereafter referred to as "vessels." Between 6 and 12 bioprinted constructs per condition were transferred into the vessels by sealing one end with parafilm (PraxisDienst) and using a sterile spoon spatula to place the constructs inside. The vessels were then filled with CO_2_-independent medium, ensuring minimal air was trapped, and sealed at the other end with parafilm. If an air bubble formed during sealing, its size was monitored to ensure it remained small enough to prevent the vessel from being divided into separate sections. The final medium volume in each vessel varied depending on the pipette trimming, the amount of trapped air, and the number of constructs included. Simultaneously, non-bioprinted spheroids (spheroid controls) were prepared by pipetting the spheroids (pre-formed in the Sphericalplate 5D) into the vessels, adjusting the total volume to 6.5 mL to achieve a final cell density of 230 000 cells/mL or around 35 spheroids per vessel. Four conditions were prepared: bioprinted 3 %, bioprinted 5 %, bioprinted 7 % and spheroid controls, this for both cell lines (MCF-7 and MDA-MB-231). Those conditions were duplicated for the 1g control (placed in the same incubator but not placed on the clinostat). The clinostat was set up to accommodate the vessels (outer diameter 7.95 mm, inner diameter 5.98 mm, 15 rpm) and was placed in an incubator at 37 °C with no CO_2_ source (Sanyo MIR-554). The samples were kept for seven days before collection for further processing – either by fixation in 4 % PFA (Millipore Sigma) in PBS, or by homogenizing the constructs and spheroids in 500 μl TRIzol (Invitrogen ThermoFisher Scientific).

### 2.6 Immunofluorescence staining

The fixed samples were incubated at room temperature for 30 min with 4 % paraformaldehyde (PFA) in PBS and 2 % PenStrep (PBS and PenStrep), washed three times in PBS with 1:500 normocin (PBS and normocin) for 5 min then left at 4 °C until immunofluorescence staining. A protocol from Loessner et al. [48] was followed. Briefly, the samples were permeabilized in Triton X-100 (0.3 % v/v, Sigma Aldrich) in PBS and PenStrep for 40 min, washed three times for 10 min with 0.1 M glycine (Carl Roth) in PBS and PenStrep and three times for 10 min with 0.1 % Triton X-100 in PBS and PenStrep (PBS-T, Millipore Sigma). The samples were then blocked for 1 h in a blocking solution comprising 0.1 % bovine serum albumin (BSA, Sigma Aldrich), 0.3 % Triton X-100 and 0.05 % Tween-20 (Carl Roth) and 10 % donkey serum (Sigma Aldrich) in PBS and PenStrep before being incubated overnight with the primary antibody diluted in blocking solution (see Supplementary Table 1). The samples were subsequently washed three times for 5 min each in PBS and 1:500 normocin before being incubated with the secondary antibody diluted in blocking solution for 2 h at room temperature and then overnight at 4 °C (see Supplementary Tables 1 and 2). After a final wash in PBS and 1:500 normocin (three times for 5 min), the objects (bioprinted or control spheroids) were transferred to a 96-well imaging plate (zell-kontakt GmbH) and imaged using a confocal microscope (Zeiss AxioObserver LSM780).

### 2.7 Quantification of proliferating cells

The channels for Ki67 and nuclei (Hoechst-stained during the previously described immunofluorescence staining) were extracted from the confocal images and the individual z-stacks were processed using Fiji by ImageJ. The intensity regions for each channel were segmented using the 3D object counter [49]. The threshold was set for each set of samples. Here, the coverage of the regions was of greater importance than the segmentation of each individual nucleus. Then, the sum segmented volume for the Ki67 channel was divided by the total segmented volume of the nuclei channel to calculate the proliferation rate.

### 2.8 Cell nuclei segmentation and counting, cell-actin ratio

The nuclei channels (Hoechst) were extracted on ImageJ and segmented using micro_sam (Segment Anything for Microscopy) [50,51]. The segmented pictures were then imported into ImageJ for 3D counting using the MorphoLibJ plugin [52] (Analyze Regions 3D – surface area method: Crofton 13 dirs and Euler Connectivity: 6). The labeled image was labeled in RGB and using the volume measure, also by MorphoLibJ. Thresholds were adjusted depending on the cell density, their proximity to one another and the intensity of the staining. The same protocol used to quantify the Ki67 to nucleus volume ratio was used to also quantify the ratio of actin to nucleus volume.

### 2.9 RNA extraction and sequencing

The samples were homogenized in TRIzol by pipetting the spheroids up and down and by using a micro pestle (Carl Roth) for the 3D bioprinted samples until the samples could be easily pipetted. All samples were processed using low-biding 1.5 ml tubes (Eppendorf) and filtered pipette tips (Nerbe Plus and Biozym). Subsequently, the samples were frozen at −20 °C until further processing. The RNA extraction followed an adapted protocol from Köster et al. [53]. The samples were thawed at room temperature and 100 μl chloroform (MP Biomedicals) was added and thoroughly mixed before incubating for 2 min at room temperature. Directly thereafter, the samples were centrifuged at 12000g for 15 min at 4 °C. The samples will separate in three phases – and the upper aqueous phase was transferred to a fresh tube before adding 250 μl isopropanol. The tubes were inverted five times and incubated at room temperature for 10 min. The samples were centrifuged again at 12000g for 5 min at 4 °C. From this point on, the samples were processed under an ultraviolet sterilizing PCR hood (Peqlab Biotechnologie). The samples were washed by removing the supernatant and pipetting 1 ml of 75 % ethanol (ThermoFisher Scientific) on the pellet before centrifuging at 7500g for 5 min at 4 °C and repeating the washing step once again. Finally, the pellet was dried for 10 min on a heating block at 50 °C (Eppendorf Thermomixer) and the dried pellet was resuspended in 12 μl DNAse/RNAse-free ultrapure water (Gibco ThermoFisher Scientific) and incubated for 10 min on a heating block at 55 °C. The concentration and purity of the RNA samples were determined using the NanoPhotometer NP80 (Implen GmbH).

RNA sequencing was conducted on a MinION Mk1B device (Oxford Nanopore Technologies) using a cDNA-PCR Barcoding kit provided by the manufacturer (SQK-PCB114.24). The concentration of total RNA used was 500 ng. The library was prepared according to the manufacturer's instructions. The samples were sequenced in triplicates using a R.10.4.1 flow cell.

The samples were basecalled and barcoded in line with the sequencing using the Nanopore MinION Mk1C software. A quality control step was additionally conducted (chosen to be above 8). The passed FastQ files were uploaded to the Epi2MeAgent software from Oxford Nanopore Technologies. The “Fastq Human Exome 2023.06.14–1867142” was used to align the reads to the human genome (GRCh38). A minimum length filter of 10 bp was implemented. A Python code adapted from Muzellec et al. [54] using the DESeq2 method [55] was used. The output log2 fold change and p value were used to determine the over expression (over 1.5) and under expression (under −1.5) of the sequenced reads. The decision was made to use the output without LFC (log2 fold change) shrinkage due to the size of the samples (between 10 491 and 345 809 reads). The volcano plots were produced using bioinfokit 2.1.4 [56]. DAVID Bioinformatics from the National Institutes of Health (NIH) [57,58] was used for gene ontology and analysis of pathways. Visualization tools from SRPlot [59] were used, such as cluster heatmap and the KEGG (Kyoto Encyclopeida of Genes and Genomes) and gene ontology (GO) enrichment analysis [60,61].

### 2.10 Image processing and statistical analysis

Experiments were repeated on three biological replicates with at least three technical replicates respectively. Images were processed using Fiji by ImageJ [62] (version1.53c, U. S. National Institutes of Health). Mathematical processing was performed on Microsoft Excel (version 16.86). Statistical analysis was conducted on Python 3.9 (Python software foundation) using the SciPy [63] and NumPy [64] packages. For data with small sample size, normality was tested with a Shapiro-Wilk test (p > 0.01) before executing the statistical comparison between two groups with a Welch *t*-test (p < 0.01). For samples with sample size bigger than 50 data points per conditions, normality was tested using a Kolmogorov-Smirnov-Test (KS-Test, p < 0.01) and statistical comparisons between groups was performed using a Mann-Whitney *U* test (p value indicated on graph). The individual p values resulting from the tests are directly written in the text when necessary. Plots were generated on Python using the Pandas [65], Seaborn [66] and Matplot [67] libraries. The boxplots were plotted so that the box ranged from the first quartile (Q1) to the third quartile (Q3) of the data, a red line being drawn for the median. The box whiskers extended by 1.5x the inter-quartile range (IQR). Additional flier points were plotted when they extended past the end of the whiskers. Graphical ﬁgures were created with Biorender.com.

## 3 Results

### 3.1 The printability and consistency of constructs produced using a high throughput low-cost bioprinter depends on the bioink

The drug development pipeline has undergone significant changes in recent years, driven by the urgency for faster delivery of effective therapeutics, as exemplified during the development of SARS-CoV-2 vaccines [68]. In response to these demands, 3D bioprinting has gained prominence in disease modeling due to its adaptability, precision, and ability to generate refined, high-throughput models that closely mimic disease conditions [69]. However, tissue engineering continues to face numerous challenges, spanning both technological and biological domains. Key issues include resolution, cell viability, and printing speed, with the latter restricting the number of objects that can be bioprinted simultaneously, thereby compromising cell health and limiting experimental replicates.

**Fig. 1:**
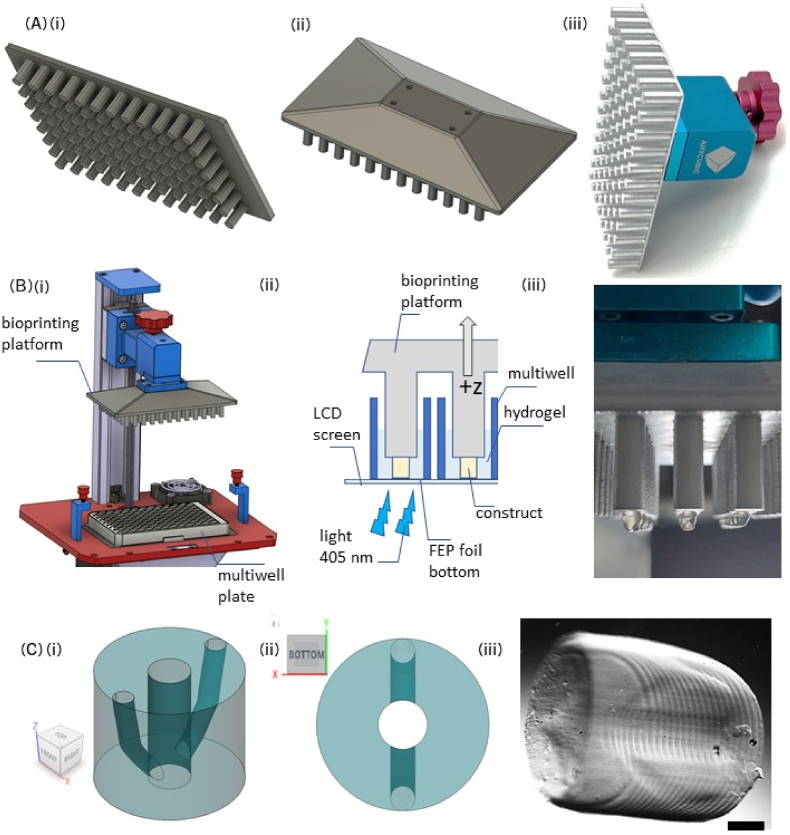
Design, Integration, and Testing of the Custom-Made 96-Well Bioprinting Platform for an Open-Source Stereolithographic (SLA) 3D Printer. (Ai-ii) A bioprinting platform consisting of 96 individual pillars, designed to fit into the wells of a 96-well plate, was created using CAD software and 3D-printed with PLA filament. (Aiii) To enhance the adhesion of bioprinted constructs and improve sterilization efficiency, the platform was coated with an aluminum spray. (Bi) The platform was designed for seamless integration with the standard z-axis holder of the open-source bioprinter (see also Aiii). When used in conjunction with a 96-well plate featuring an FEP-foil bottom, the platform enables high-throughput bioprinting of 96 constructs in a single run. Modified from https://grabcad.com/library/photon-3d-printer-assembly-1. (Bii) A diagram illustrating the bioprinting process. A commercially available 96-well plate with an FEP-foil bottom is positioned on the LCD screen of the bioprinter. Blue light at 405 nm back-illuminates the LCD screen and patterns the photopolymerization of the bioink in correspondence of the pillars. The platform is progressively raised along the z-axis printing layer-by-layer the desired construct. (Biii) Photograph of GelMA-based constructs on the pillar tops after bioprinting. (Ci-ii) CAD rendering of a bioprinted construct featuring branched channels, side view and top view, respectively. (Ciii) Magnified view of a single bioprinted cylinder under dark-field contrast, revealing the central channel and two laterally branching channels. The broad base of the construct is a result of the longer exposure time applied to the initial bottom layers to ensure stable adhesion to the pillar's top. This extended exposure time creates a thick over-polymerized substrate, which is accounted for in the construct's design. The GelMA concentration used for this construct was 7 %. Imaging details: Microscope: Zeiss SteREO Discovery V8 Objective: Plan Apo S, 0.63x, FWD 81 mm, Camera: AxioCam IcC SIN.Pixel size: 4.54 × 4.54 μm^2^, Scale bar: 500 μm. (For interpretation of the references to color in this figure legend, the reader is referred to the Web version of this article.)

To overcome this limitation, a platform compatible with standard 96-well plates was designed and fabricated (see Fig. 1Ai-iii). In SLA bioprinting, the platform serves as the substrate where bioprinted objects adhere during the printing process. To enhance sterilization and improve the adhesion of the bioink to the platform during the photopolymerization process, the platform was coated with an aluminum layer. This coating serves two key purposes: first, it smooths the surface of the platform, eliminating grooves caused by the filament-based additive manufacturing process, which are difficult to sterilize effectively. Second, it ensures strong adhesion of the initial layers of the 3D bioprinted structure, which is critical for successful bioprinting. Without the aluminum coating, the polylactic acid (PLA) thermoplastic material of the platform does not provide sufficient adhesion strength. Fig. 1A illustrates the platform design (i-ii) and the actual platform after the aluminum coating process (iii). The procedure is conducted in an inverted configuration, with the platform submerged in bioink contained within individual wells of a 96-well plate (Fig. 1 Bi-ii and Supplementary Fig. 1 Bi). The bioprinted construct is fabricated layer by layer, with the initial layer projected onto the surface of each mini platform. Following the curing of each layer, the platform moves incrementally upward, repeating the process until all layers are successfully printed, resulting in the final 3D construct (Fig. 1 Bii). The addition of 96 mini-platforms, resulting in 96 objects being printable at once, facilitates high-throughput capabilities, significantly increasing the number of replicates that can be generated in a single experiment (Supplementary Fig. 1 Bii). The enhanced throughput not only improves experimental efficiency but also enables the attainment of statistical significance, a critical requirement in the drug development pipeline for robust and reliable results. The user could also potentially bioprint 96 different cell types or models at once, increasing the testing capabilities. A photosensitive bioink, defined as a solution of one or more biomaterials in hydrogel form, typically encapsulating the desired cell types, was utilized as the liquid precursor that will be crosslinked by the projected light of the bioprinter. The custom-made platform was integrated with a modified Anycubic bioprinter, described in earlier work [29]. This modified SLA 3D printer, equipped with temperature and CO_2_ controls, provided an economical and efficient option for disease modeling.

The new platform was tested using different formulations of hydrogel. The hydrogel used for the bioink was a mixture of gelatin methacrylate (GelMA) and poly(ethylene) glycol diacrylate (PEGDA) at various ratios (3%–3%, 5%–3% or 7%–3% w/v GelMA-PEGDA respectively). Those ratios were used to model different stiffness, the stiffness increasing with the GelMA content [70,71]. GelMA, being cell-degradable, could easily be modulated and was combined with PEGDA that ensures mechanical support and structural integrity [16]. GelMA is animal-based and might not be as well-defined as other matrices but is easily synthesized and therefore low-cost [72]. The mechanical properties and therefore the stiffness of the hydrogel ratios was measured in a previous study using rheology – indeed, rheology provides dynamic insights in hydrogels’ behavior (shear stress), which is more representative of the physiological conditions. In this previous work, 3%–3% was measured at around 1500 Pa, 5%–3% at around 2800 Pa and 7%–3% at around 4200 Pa [29]. Hereafter, the ratios will be referred to as 3 %, 5 %, and 7 %.

Initially, printability was assessed without adding cells to the hydrogel mixture. The bioprinted object selected for this study was in the shape of a cylinder with branching channels. The CAD design of the 3D structure is shown in Fig. 1 Ci-ii. The cylinder was selected as a physiologically relevant shape (for example representing the mammary ducts) and the channels would ensure nutrient medium delivery to cells within the constructs. Various hydrogel compositions were printed randomly on some of the platforms in the 96 array to verify the functionality of all wells (Supplementary Fig. 1 A). The prints took approximately 37 min to complete, after which the resulting objects were observed under a phase contrast microscope (Fig. 1 Ciii). The primary and side channels were clearly identifiable post-printing and washing (Fig. 1 Ciii and Supplementary Fig. 1 Cii, red circle and lines and yellow lines), although a thin layer could cover the bottom of the channels due to overexposure during printing of the initial layer. Supplementary Fig. 1 Ci shows the construct in the context of a well in a 96 well-plate.

Top-view imaging and diameter measurement of the objects confirmed print quality (Supplementary Fig. 1D). The designed CAD dimensions were 3.5 mm in diameter and 3 mm in height (Fig. 1 Ci). Measured diameters of the printed cylinders varied slightly with hydrogel concentration (Supplementary Fig. 1 Di), but no significant differences were detected among the groups (p = 0.83 between 3 % and 5 %, p = 0.49 between 5 % and 7 %, and p = 0.38 between 3 % and 7 %). The large error bars further indicated a variability in diameter variability as a result of the device's inherent inaccuracy. Microscopic observations (Supplementary Fig. 1 Dii) revealed that the 3 % cylinders were less defined compared to the 5 % and 7 % constructs. However, the channels were clearly defined after bioprinting as visible in phase contrast images (Fig. 1 Ciii). As reported previously [29], GelMA/PEGDA hydrogels tend to swell by 1.5 to 2-fold after approximately 24 h in medium or PBS, which would help compensate for the smaller-than-designed diameter (the constructs were imaged in PBS after an extensive wash, in which case the swelling would not be entirely completed). We demonstrated that the modified Anycubic bioprinter, while not achieving the highest design precision, offered a satisfying cost-effective alternative.

The successful operation and printability of the adapted 3D bioprinter with the newly designed 96-well plate platform enabled further experimentation with cell-encapsulated hydrogels to form bioinks for 3D bioprinting.

### 3.2 The stiffness of the matrix affects the metabolism of bioprinted breast cancer cells

Bioprinting can have a pronounced negative effect on the cells depending on the technique used. Cells embedded in the bioink before bioprinting can be affected by the chemistry of the bioink and the bioprinting technique employed [[73], [74], [75]]. Therefore, it is crucial to assess how the cells react to the bioprinting method of interest before conducting further experiments.

MCF-7 cells were used for cell viability and metabolism measurements. The MCF-7 cell line, derived from an adenocarcinoma of the mammary gland, was established from a pleural effusion from a 69-year-old Caucasian female. These cells express a range of crucial receptors (estrogen receptor alpha, as well as androgen, progesterone, and glucocorticoid receptors), making this cell line attractive for medical research [76]. MCF-7 cells are classified as luminal A ER+, PR+/−, HER2- [77].

**Fig. 2:**
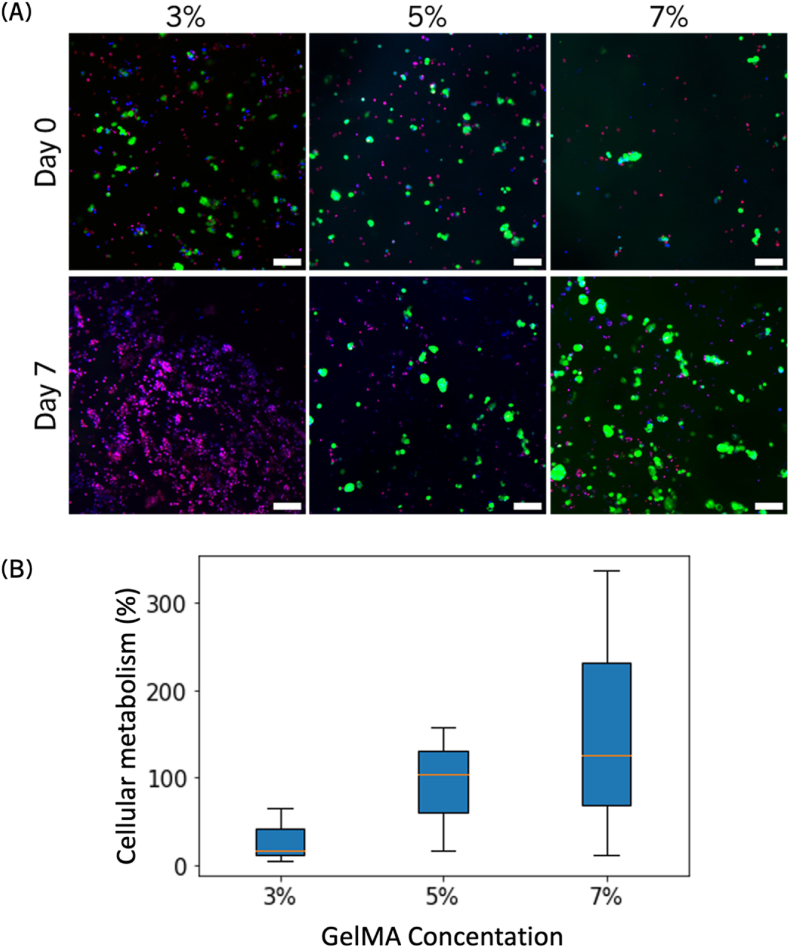
The cell viability of the MCF-7 cells was affected by the stiffness of the ECM for hydrogels composed of 3%–3%, 5%–3% or 7%–3% w/v GelMA-PEGDA, referred to as 3 %, 5 % and 7 % respectively. (A) Live dead assay staining of the MCF7 spheroids directly after bioprinting (day 0) and after seven days in a CO_2_ independent medium (day 7) for various ECM stiffnesses. The live cells were stained using FDA (in green), the dead cells using PI (in red) and the total nuclei with Hoechst 33342 (in blue). Scale bar: 50 μm. (B) Results of the CTG assay measuring the ATP consumption after 7 days in a CO_2_ independent medium, normalized to the results on day 0 for various hydrogel stiffness (n = 3). (For interpretation of the references to color in this figure legend, the reader is referred to the Web version of this article.)

Spheroids of MCF-7 cells were first formed in specialized well-plates for two to four days before encapsulating the spheroids in the hydrogel and bioprinting. Spheroids are three-dimensional models consisting of cells self-aggregating into a compact sphere [47,78]. This compaction mimics the environment of the cells within a tumor and spheroids are therefore a well-used model in cancer research and oncology drug screening [79]. Cells were stained with FDA (for live cells) and PI (for dead cells), with all cell nuclei stained with Hoechst 33342. Imaging was performed immediately after bioprinting (day 0) and after seven days in a CO_2_-independent medium (day 7) (Fig. 2 A). Immediately after bioprinting, a mixture of live and dead cells was observed in all conditions, with a majority of live cells. The presence of dead cells could be attributed to radicals in the bioink, such as the cleaved photoinitiator, LAP. After seven days, very few live cells were detected in the 3 % bioink, whereas live cells in the 5 % and 7 % bioinks appeared to have proliferated.

Quantification of adenosine triphosphate (ATP) levels produced by live cells on day 0 and day 7 confirmed these observations (Fig. 2 B). ATP is a co-factor required for many essential biochemical reactions, and its cleavage produces high levels of energy, making it the “fuel” of cells [80,81]. The amount of ATP produced is therefore a direct correlation with the cell metabolism: an increase of ATP production indicates that the cells are thriving, whereas a decrease signifies senescence. It should be noted that MCF-7 cells are naturally ATP-high which rendered them particularly suited for ATP-derived studies. Cells in the 3 % bioink showed very little increase in ATP on day 7 compared to day 0 (29 % on average). In contrast, the 5 % hydrogel bioink demonstrated steady ATP consumption (average of 93 %), while the 7 % bioink showed a significant increase (158 %). The increase in ATP consumption is reflected by the increase in FDA-positive, live cells in the 5 % and 7 % condition whereas cell death in the 3 % condition is also shown in lower ATP consumption after 7 days relative to the cells immediately after bioprinting. This indicated that the 3 % hydrogel did not support MCF-7 cell expansion and proliferation, whereas the 5 % and 7 % hydrogels promoted steady to robust cell proliferation.

These results confirmed that the stiffness of the BC microenvironment plays a crucial role in cell proliferation and viability, consistent with findings from previous studies [82]. As a comparison, it was shown that the elastic modulus of the healthy mammary gland was measured at 167 Pa whereas the average tumor was measured upward from 4000 Pa [83], in which case the stiffness chosen for the bioprints represented a very early onset of the disease (perhaps less suitable to this particular cell line, as shown by the low cell viability) up to the stiff diseased microenvironment. Indeed, the shear complex modulus of objects printed with 3 %, 5 % and 7 % mixtures were previously measured to be ranging from 1500 to 4200 Pa, as quantified by rheometer, a well-suited tool to measure viscosity as well as viscoelasticity of hydrogels [29]. Therefore, the cell viability and metabolism results indicated that a stiffer environment better supported the growth and activity of aggressive cancerous cell lines compared to a softer one, reflecting a physiologically relevant condition for breast cancer cells. The effect of extracellular matrix stiffness on specific markers in BC cells was subsequently evaluated.

### 3.3 Nuclear markers for breast cancer proliferation show varied results

ECM stiffness, also known as ECM stress, has been shown to influence cell morphology, proliferation, and metastasis in BC cells [27,[84], [85], [86], [87], [88]]. The progressive increase in matrix stiffness associated with tumor progression, linked to collagen accumulation, is a process referred to as "desmoplasia" [27]. During BC progression, the stiffness of the tumor microenvironment can increase up to 24-fold compared to the healthy breast tissue [89]. Consequently, models representing more advanced stages of BC—those correlated with a poorer prognosis [90]—must account for a stiffer microenvironment. To address this, MCF-7 spheroids were encapsulated in matrices of varying stiffness and subsequently subjected to simulated microgravity (referred to as 0g) for 7 days to analyze the expression of proteins relevant to proliferation and ECM interaction through immunofluorescence. Parallelly, it has been shown in single cell culture of colorectal cancer under simulated microgravity that Yes-associated protein (YAP) was activated in microgravity, leading to an increase in autophagy-related pathways [91]. Combining the impact of the ECM and simulated microgravity could help elaborate on which effect has the most influence.

**Fig. 3:**
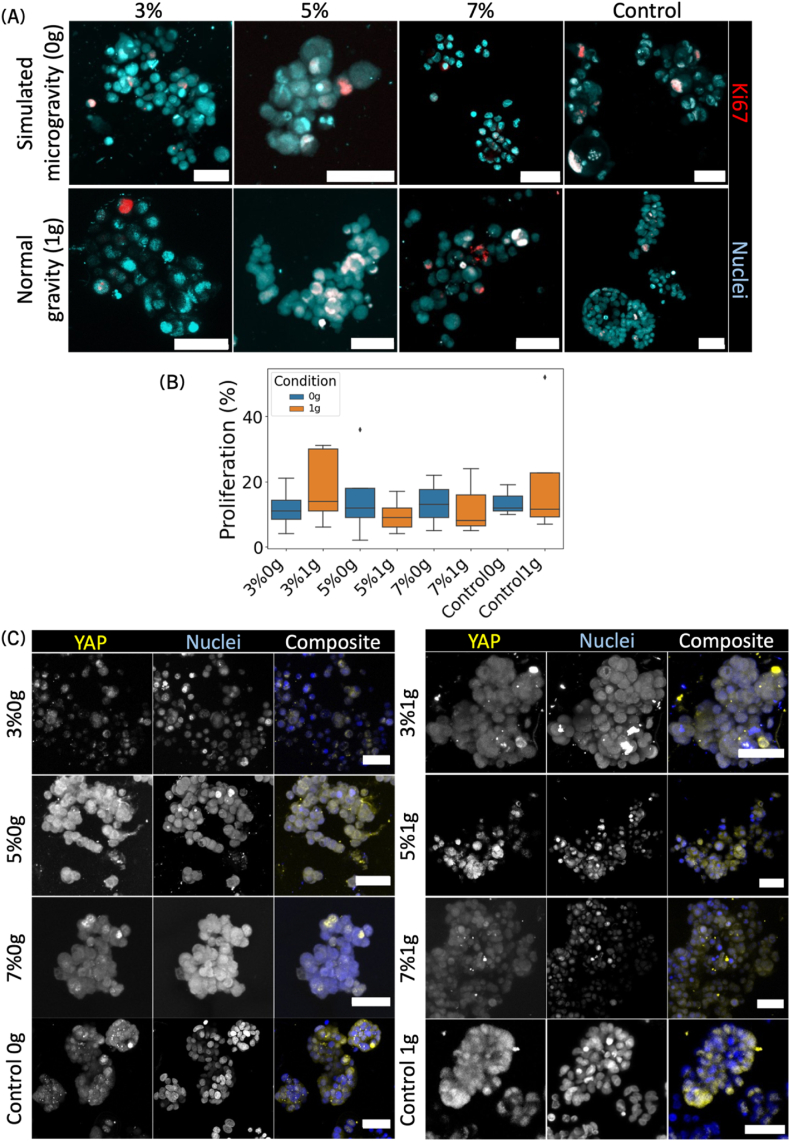
The stiffness of the ECM did not influence cell proliferation but played a significant role in the relocation of mechanotransducers. (A) Representative immunofluorescent images of Ki67 (a proliferation marker, shown in red) and Hoechst (nuclei, shown in cyan) for samples bioprinted in matrices with increasing stiffness (3 %, 5 %, and 7 %), along with non-printed spheroid controls. These samples were cultured under simulated microgravity (0g) and earth gravity (1g) conditions. (B) Quantification of proliferating cells under all conditions is depicted. For instance, "3 %0g" indicates a hydrogel composition of 3 % GelMA/3 % PEGDA cultured under simulated microgravity. Proliferation was calculated by comparing the volume of proliferating cells to the volume of nuclei within each sample (n = 3 to 5). (C) Representative images of immunofluorescent staining for YAP (a mechanotransducer marker, shown in yellow) and Hoechst (nuclei, shown in cyan) are provided for all samples. The data presented in (A) and (C) are from one experiment that is representative of three biological replicates and three technical replicates. Microscope: Zeiss AxioObserver LSM780. Objective: Plan ApoChromat 20 × /0.8 M27. Scale bars = 50 μm. (For interpretation of the references to color in this figure legend, the reader is referred to the Web version of this article.)

The proliferation marker Ki67 was first examined. Encoded by the MKI67 gene, Ki67 is expressed during all active phases of the cell cycle, peaking during the M phase [92]. Since proliferation is a critical indicator of cancer aggressiveness and tumor progression, Ki67 has also been recognized as a predictive marker for therapeutic responses [93,94], making it a key target for investigation. Staining for Ki67 and nuclei was performed, followed by imaging and quantification of the proliferation rate. No significant visual differences were observed between the samples, and the quantification yielded no statistically significant differences in proliferation rates (Fig. 3, Welch *t*-test with α = 0.05, n = 3 to 5).

Subsequently, the focus was shifted to YAP. YAP, often associated with TAZ (a transcriptional coactivator with a PDZ-binding motif), responds to ECM rigidity and shape by localizing either to the cytoplasm (in softer ECM) or to the nucleus to activate genes related to cytoskeleton remodeling and cell proliferation (in stiffer ECM) [[95], [96], [97]]. In printed constructs subjected to 0g, YAP was increasingly localized to the nucleus, whereas in the control non-printed spheroids, the opposite pattern was observed. Notably, the leading "buds" of the spheroids in the printed constructs exhibited particularly intense nuclei YAP signaling (see 5 %0g and 7 %0g). In earth gravity (1g), YAP predominantly localized to the cytoplasm, both in printed spheroids and in controls. A comprehensive overview of YAP, Ki67, actin and nuclei staining are provided in Supplementary Fig. 2.

Following the examination of the mechanotransducer YAP, the study proceeded to investigate the epithelial-to-mesenchymal transition (EMT) using the markers EpCAM and Vimentin.

### 3.4 Epithelial-to-mesenchymal transition occurs at 1g but not in simulated microgravity

The phenomenon of epithelial-to-mesenchymal transition (EMT) occurs in BC cells as they lose their epithelial characteristics and acquire mesenchymal properties, enabling them to detach from the primary tumor and become metastatic [41,98]. Epithelial cell adhesion molecule (EpCAM) is a transmembrane protein responsible for cell-to-cell adhesion [99]. It has been considered a marker for epithelial tumors, typically downregulated in mesenchymal tumors [100,101]. Conversely, vimentin, a type III intermediate filament protein, plays a crucial role during cell migration [102] and is pivotal during EMT [103,104]. The loss of EpCAM, coupled with an increase in vimentin, is generally indicative of EMT and is associated with more aggressive BC phenotypes [101,105]. This study utilized two BC cell lines: the less invasive MCF-7 and the highly aggressive MDA-MB-231 [77,106]. MCF-7 cells are characterized as luminal A ER+, PR+/−, HER2-, while MDA-MB-231 cells are claudin-low and triple-negative [77]. A phase-contrast image of the cells is provided in Supplementary Fig. 3.

**Fig. 4:**
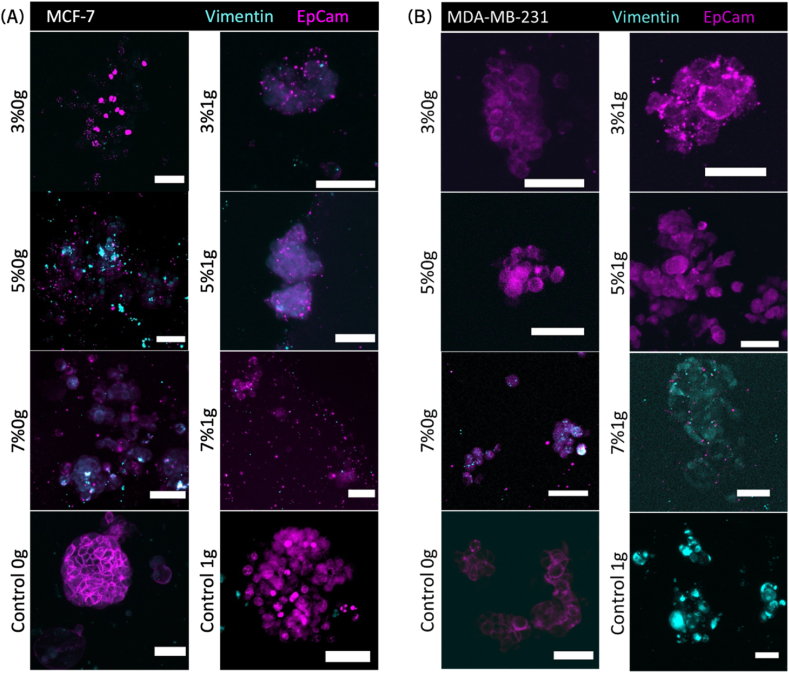
Stiffness influenced the expression of EMT markers, EpCAM (epithelial marker) and vimentin (mesenchymal marker) in BC, whether in both less aggressive (MCF-7 (A)) or highly invasive (MDA-MB-231 (B)) cell lines. The samples were bioprinted in matrices with increasing stiffness (3 %, 5 %, and 7 %), along with non-printed spheroid controls. These samples were cultured under simulated microgravity (0g) and normal gravity (1g) conditions, then stained against vimentin in cyan and EpCam in magenta. The data is from one experiment that is representative of three biological replicates and two technical replicates. Microscope: Zeiss AxioObserver LSM780. Objective: Plan ApoChromat 20 × /0.8 M27. Scale bars = 50 μm. (For interpretation of the references to color in this figure legend, the reader is referred to the Web version of this article.)

Spheroids from both cell types were printed in the different hydrogel compositions and incubated in 1g or 0g for seven days prior to fixation and immunofluorescent staining for vimentin and EpCAM. Non-printed spheroids were used as control. The results are presented in Fig. 4. Single-channel images and merged images, including actin and nuclei staining, shown in Supplementary Figs. 4 and 5.

The MCF-7 cells predominantly expressed EpCAM; however, vimentin expression was also observed, faintly at lower matrix stiffnesses (3 % and 5 %) in 1g and localized at higher stiffness (5 % and 7 %) in 0g conditions (Fig. 4 A). The control samples did not exhibit any vimentin expression, maintaining a clear EpCAM pattern. In contrast, the MDA-MB-231 cells exhibited a more distinct pattern (Fig. 4 B). Softer ECM (3 % and 5 %) under both 0g and 1g conditions showed clear EpCAM expression, while stiffer environments (7 %) under 1g conditions displayed pronounced vimentin expression. In simulated microgravity, vimentin expression was still present but less prominent. The control samples also showed vimentin expression under 1g, whereas no vimentin expression was detected under 0g. In both cases, the expression of EpCAM and vimentin were reversed in 0g. This indicated the occurrence of EMT in 1g for higher stiffness and no ECM at all, and EMT was reversed in 0g. This was also the findings of Filiz, Arslan et al. [107].

The observation that the nuclei of cells encapsulated within bioprinted constructs were larger than those of the control samples prompted a detailed investigation into nuclear size and the nucleus-to-cytoplasm (N/C) ratio. These parameters were further analyzed to understand the potential implications of these morphological changes in the context of cellular behavior and response to the bioprinted microenvironment.

### 3.5 Nuclei size varies according to the stiffness of the ECM

It has been shown that a change in nucleus size in cancer cells correlates with poorer diagnostic [[108], [109], [110]]. Indeed, in most tumors, the nuclear to cytoplasmic or karyoplasmic ratio (N/C) is disrupted, as the nucleus increases in volume [109,111]. Here, we investigate whether the various conditions influence the nucleus size and karyoplasmic ratio.

**Fig. 5:**
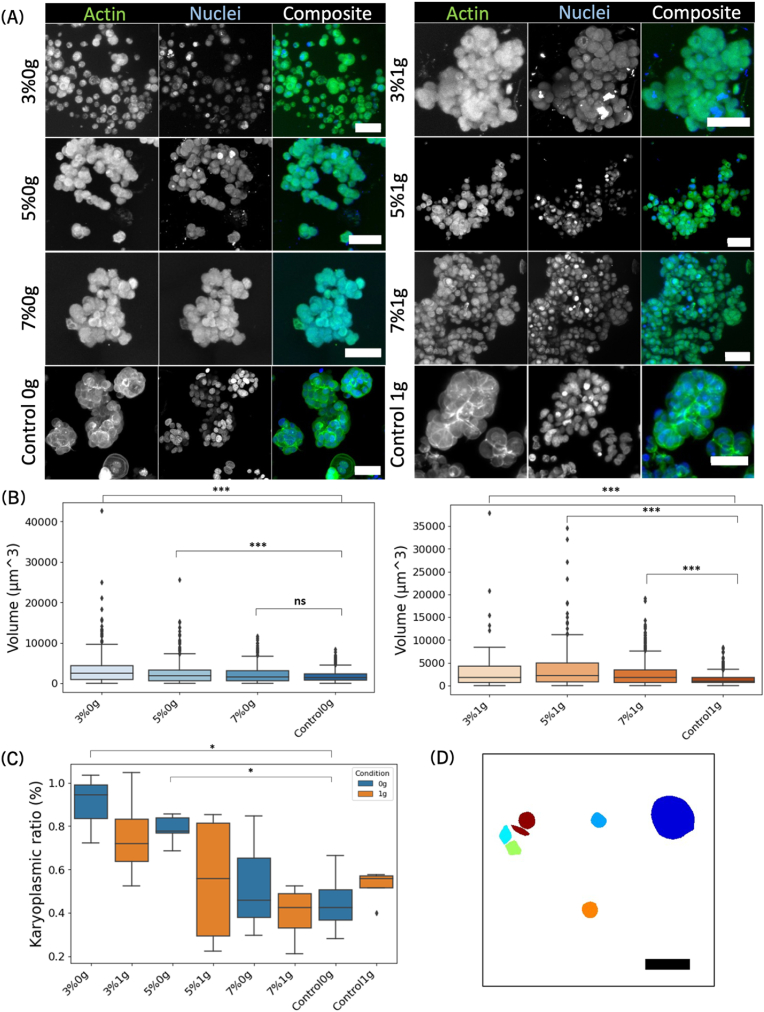
The size of the nucleus as well as the karyoplasmic ratio correlate with cancer aggressivity. (A) Representative pictures of immunofluorescent staining of MCF-7 spheroids stained with phalloidin for actin (green) and Hoechst 34580 for the nuclei (blue). The data is from one experiment that is representative of three biological replicates and five to six technical replicates. Microscope: Zeiss AxioObserver LSM780. Objective: Plan ApoChromat 20 × /0.8 M27. Scale bars = 50 μm. (B) Plot of the nuclei volumes for samples grown in simulated microgravity (0g, left) and earth gravity (1g, right). The samples showed a significant decrease compared to their respective controls (3 %0g: p = 4.35e-18, 5 %0g: 4.03e-4, 3 %1g: p = 6.71e-8, 5 %1g: p = 2.60e-18, 7 %1g: p = 4.76e-13). The samples were tested for normality with a Kolmogorov-Smirnov test. The data is tested for significance with a Mann-Whitney *U* test, n = 128 to 704, p < 0.005 (∗∗∗), p < 0.01 (∗∗) and p < 0.05 (∗). (C) Karyoplasmic ratio of the bioprinted samples grown in 0g and 1g compared to their respective controls. The lower stiffnesses (3 % and 5 %) were significantly different from their controls (3 %0g: p = 0.036, 3 %1g: p = 0.032 and 5 %0g: p = 0.014). The samples were tested for normality with a Shapiro-Wilk test. The data is tested for significance with Welch's *t*-test, n = 3 to 5, p < 0.005 (∗∗∗), p < 0.01 (∗∗) and p < 0.05 (∗). (D) Representative picture of the segmented nuclei (single frame) for 3 %1g. Scale bar: 50 μm. (For interpretation of the references to color in this figure legend, the reader is referred to the Web version of this article.)

It was visually assessed that the morphology of the cells looked different in different conditions. Indeed, the lower stiffness conditions (3 % and 5 %) showed bigger nuclei than higher stiffness (7 %) and the controls, irrespective of whether they were cultured in earth gravity or simulated microgravity (see Fig. 5 A). To confirm this visual inspection, the nuclei in each sample were segmented and quantified and their volumes were plotted in Fig. 5 B. The size of the nuclei was significantly bigger for nearly all samples compared to their respective control (p < 0.01), except for 7 %0g (p = 0.053). It should be noted that the sizes of the nuclei were quite concentrated around the mean, except for a few very large outliers, mirroring the heterogeneity of the samples (see Fig. 5 D). The higher stiffness (7 %) and controls (for both 0g and 1g) were more homogenous as well as being smaller, showing that in those conditions, nuclei grew normally. Taken together, we showed that the bioprints had nuclei of larger volumes compared to the control in 0g and 1g, except for 7 %0g, indicating that microgravity reversed the aggressivity of the cancer in higher stiffness.

Next, to understand whether only the nucleus had swollen or whether the entire cell was growing, the karyoplasmic ratio was investigated. Indeed, both the size of the nucleus progression [[112], [113], [114]] as well as cytoplasm to nuclear ratio [115,116] have been used to assess BC progression. The total volume of the nuclei as well as the total volume of the actin cytoskeleton were measured and their ratio to one another was plotted in Fig. 5 C. The higher the ratio, the bigger the nucleus was compared to the total volume of the cell. For the softer conditions (3 %0g, 3 %1g and 5 %0g), the ratio was close to one, showing that there was nearly no cytoplasm left in the cells. When comparing the same stiffness cultured in different gravitational conditions (3 %1g and 3 %0g for example), no significant difference was identified. However, there was a significant difference between the bioprinted constructs and their respective controls for 3 %0g (p = 0,036) and 5 %0g (p = 0,014) versus control 0g. It should be also noted that some samples showed a large variance (see Supplementary Fig. 6), especially 3 %1g, 5 %1g and 3 %0g. These results indicated that the tested conditions not only increased the mean nuclear size but also resulted in highly variable nuclear sizes. The variability decreased with increasing stiffness and was lowest in the controls, which exhibited the least deviation from the mean. Under 1g conditions, the mean nuclear sizes were generally larger and more variable compared to those under 0g conditions.

A larger karyoplasmic ratio was then noticed in 0g for softer matrices compared to 7 % and controls, an effect that was not present in 1g. Therefore, we demonstrated that the size of the nuclei was significantly bigger for softer bioprinted conditions than for higher stiffnesses and controls. The cytoplasm-to-nucleus or karyoplasmic ratio confirmed that the softer conditions had higher ratios than the stiffer conditions and the controls, but with less certainty due to large error bars. The substantial heterogeneity in the data reflected a high variability among the cells within each sample.

### 3.6 RNA sequencing followed by differential gene expression show that simulated microgravity modulates the effect of the ECM

Traditionally, differential gene expression analysis has been performed using real-time quantitative polymerase chain reaction (RT-qPCR). This method, however, is inherently limited by the requirement to pre-select a specific set of genes for analysis, typically constrained by budgetary considerations, which can introduce significant bias into the results [117]. In comparison, RNA sequencing offers a comprehensive, unbiased view of the entire transcriptome, facilitating the identification of novel gene expression patterns. For our study, we employed the Oxford Nanopore Technologies MinION platform to sequence RNA from bioprinted constructs composed of 7 % hydrogel, as this condition exhibited the most pronounced changes in previous experiments. Constructs were subjected to simulated microgravity (0g) and earth gravity (1g), while controls consisted of spheroids cultured in liquid environments under both conditions. Differential gene expression was analyzed using DESeq2.

**Fig. 6:**
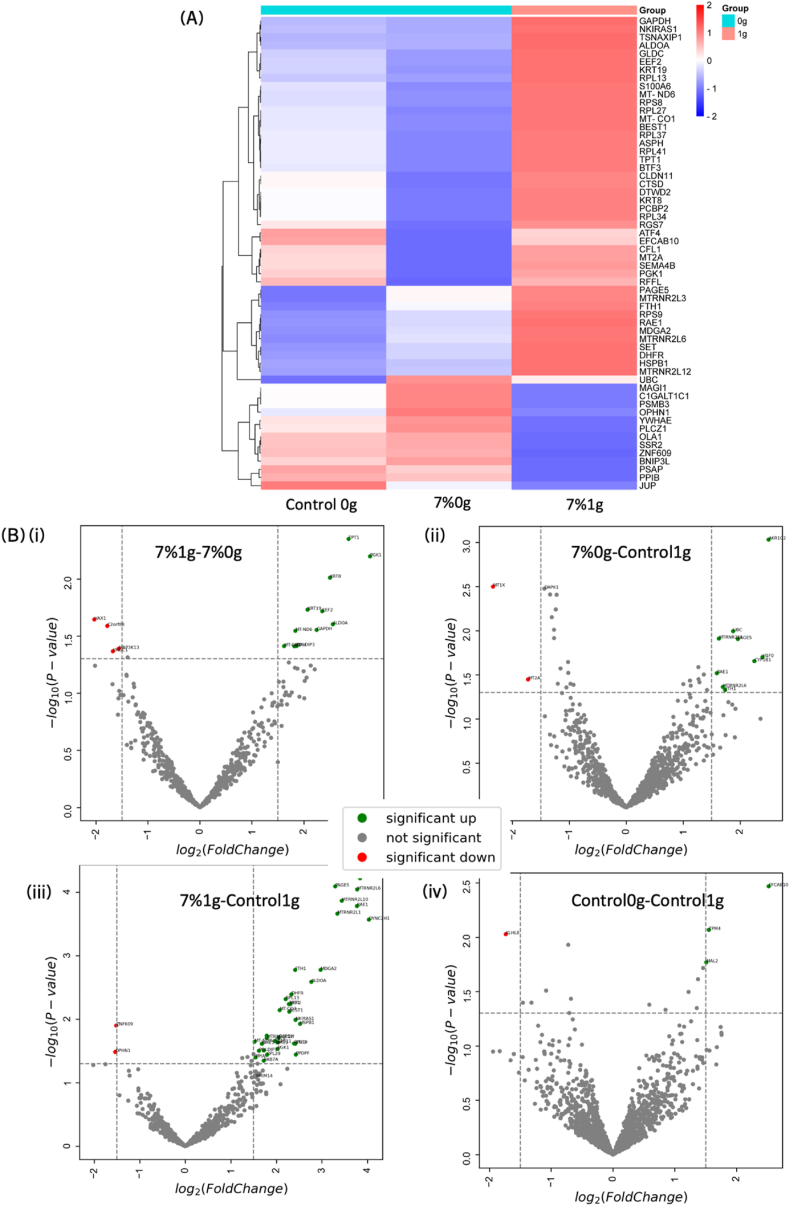
RNA sequencing of bioprinted constructs and spheroid controls indicated which genes have been over or underexpressed in differential expression between conditions, highlighting pathways and genes of interest for potential therapeutic applications. (A) Heatmap of selected genes for conditions 7 %1g, 7 %0g and control 0g compared to control 1g. The scale from red to blue shows the fold change as determined using DESeq2. (B) Volcano plots showing the distribution of differentially expressed genes between over- (more than zero) and underexpressed (less than zero). The statistically significant genes are marked in green (overexpressed) and red (underexpressed) as calculated using a Wald-test. Significance for fold change was set at 1,5 and for p-value at 0,05. The following conditions were compared: (i) 7 %0g and 7 %1g, (ii) 7 %0g and control 1g, (iii) 7 %1g and control 1g, (iv) control 0g and control 1g. (For interpretation of the references to color in this figure legend, the reader is referred to the Web version of this article.)

As seen on Fig. 6 A, sequenced genes for the bioprinted constructs (7 %0g and 7 %1g) as well as control 0g were compared to control 1g and plotted on a heatmap. There were no similar expression patterns for all three comparisons (all three conditions were up- or downregulated for the same gene).

Some genes, for example glyceraldehyde-3-phosphate dehydrogenase (GAPDH), Keratin 19 (KRT19) and Translin Associated Factor X Interacting Protein 1 (TSNAXIP1) were downregulated in the 0g conditions but upregulated in the 7 %1g hydrogel constructs compared to control 1g, indicating that simulated microgravity reversed the expression pattern for those genes regardless of matrix conditions. It was shown that the expressions of these genes were increased in invasive and metastatic BC, although TSNAXIP1 was not prominent in the literature [[118], [119], [120]]. MT-RNR2-Like 3 and 6 (MTRNR2L3/6) and Ribonucleic Acid Export 1 (RAE1), which are less well-defined in the context of BC but generally linked to tumor aggressiveness [121,122], were also upregulated in 7 %1g compared to control 1g but downregulated in the 0g conditions. Notably, MAM Domain Containing Glycosylphosphatidylinositol Anchor 2 (MDGA2) also showed the same differential expression but was only studied in gastric cancer [123] and keratin 8 (KRT8), less expressed in proliferating BC cells, was also present in this group [124]. Finally, identified genes that were upregulated in 7 %1g but downregulated in the 0g conditions were related to apoptosis and the NF-kB pathway (Heat Shock Protein Family B (Small) Member 1 (HSPB1) and MT-RNR2 like 12 (MTRNR2L12)) [125,126].

In contrast, genes like Prosaposin (PSAP), Zinc Finger Protein 609 (ZNF609) and Obg-Like ATPase 1 (OLA1), linked to chemoresistance, metastasis and poor prognosis, were downregulated in the 7 %1g constructs but upregulated in the 0g conditions [127,128]. This downregulation in 1g compared to 0g could indicate a more complex role of these genes than previously investigated.

Some other genes were downregulated in 7 %0g but not in control 0g and 7 %1g, showing that the addition of ECM and simulated microgravity triggered a change in gene expression: Metallothionein 2A (MT2A), EF-Hand Calcium Binding Domain 10 **(**EFCAB10) or Phosphoglycerate Kinase 1 (PGK1) [[129], [130], [131]]. Those genes were upregulated in aggressive types of BC, although the role of EFCAB10 was less established in BC.

The occurrence of EMT was confirmed under 7 %1g and control 1g conditions through the observation of EpCAM (see 3.4) but was absent in 0g conditions. Gene sequencing further revealed significant EMT-related changes. In control 0g compared to control 1g, Interferon alpha-inducible protein 27 (IFI27) and Annexin A1 (ANXA1), typically overexpressed during EMT in cancers [132,133] were downregulated (log2 fold changes of −1.458 and −1.174, respectively), suggesting that microgravity may reverse EMT in free-floating spheroids, as shown by previous studies [107]. Additionally, under 7 %0g compared to 7 %1g, Keratin 8, 18, and 19 (KRT8, KRT18, KRT19), which are usually underexpressed during EMT in cancers [[134], [135], [136]], were also downregulated (log2 fold changes of −2.504, −1.732, and −2.072, respectively), indicating that the stiff ECM's effect on EMT was negated under microgravity conditions. Individual genes were plotted for comparison of conditions in volcano plots (Fig. 6 B). First, we noticed that the differential gene expression between all conditions yielded more significantly upregulated genes than significantly downregulated (fold change 1.5 or −1.5 respectively), showing a clear activation of pathways when ECM was involved, especially in simulated microgravity. Interestingly, there was no overlap of significantly over- or underexpressed genes between 7 %0g and 7 %1g compared to control 1g (Fig. 6 B ii and iii). Minimal differences were observed between the 0g and 1g controls, with only a few significantly regulated genes (only 3 significantly upregulated genes and 1 significantly downregulated genes). Focusing on the comparison between 7 %1g and 7 %0g, the genes significantly upregulated were related to cancer aggressiveness, proving here again that the presence of simulated microgravity reversed the effect of the stiff ECM.

**Fig. 7:**
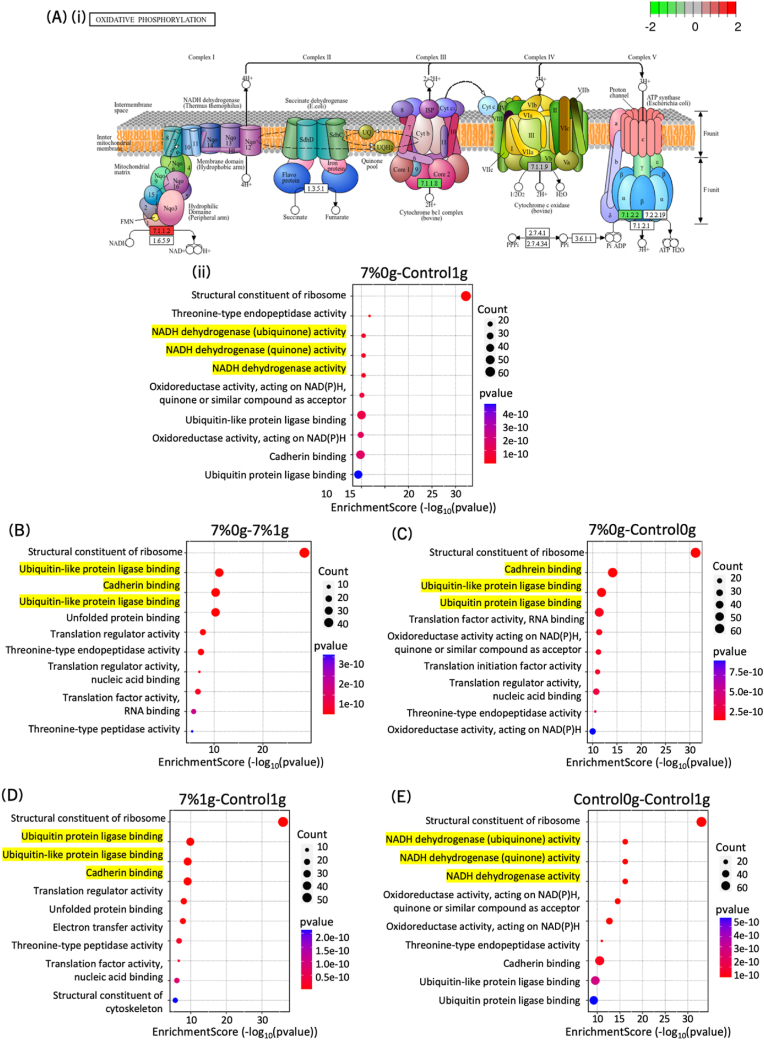
KEGG (Kyoto Encyclopedia of Genes and Genomes) pathway and gene ontology enrichment score results for molecular function. The differential gene expression between two conditions is shown: (A) (i) 7 %0g and control 1g, (ii) showing the impacted genes within the KEGG pathway “oxidative phosphorylation”, (B) 7 %0g and 7 %1g, (C) 7 %1g and control 0g, (D) 7 %0g and control 1g and (E) control 0g and control 1g. The important functions are highlighted in yellow. (For interpretation of the references to color in this figure legend, the reader is referred to the Web version of this article.)

Gene ontology (GO) enrichment analysis for molecular function was plotted (Fig. 6). Mostly structural constituents of the ribosome were enriched, which related to the ribosomal RNA that was analyzed during RNA sequencing but has no impact on the gene expression. There was an increase in NADH dehydrogenase activity in the 7 % hydrogel constructs under 0g conditions (Fig. 7 A) and in the 0g control (Fig. 7 E) relative to the 1g control, suggesting enhanced oxidative phosphorylation linked to drug resistance in cancer cells [137] (see genes involved in Fig. 7 A ii). Additionally, enhancement of the activity of the mitochondrial complex I through NAD+/NADH redox balance and autophagy has been shown in BC metastasis [138]. The comparison between 7 %1g to the control 1g, 7 %0g to the control 0g as well as 7 %0g to 7 %1g showed enrichment in two pathways: ubiquitin and ubiquitin-like protein ligase binding and cadherin binding (Fig. 7B and C and D). The contrast between 7 %0g and control 0g on one hand, and 7 %1g and control 1g on the other showed a more enhanced cadherin binding for the former and a more enhanced ubiquitin(-like) protein ligase binding for the latter. The ubiquitin-proteasome system is an important factor in autophagy. It regulates BC progression through EMT regulation and microenvironment remodeling [139,140] (Supplementary Fig. 7C). Finally, cadherin binding is a crucial part of the adherent junction, involved in EMT and leading to metastasis when downregulated (Supplementary Fig. 7 D). Finally, 7 %0g compared to 7 %1g showed a combination of the ubiquitination and cadherin binding pathways (Fig. 7 E).

## 4 Discussion

Breast cancer manifests in various forms, including basal, luminal, and triple-negative subtypes, with the more aggressive variants posing significant treatment challenges, particularly when metastasized [141]. Metastasis occurs when cancer cells disseminate from the primary tumor through the blood or lymphatic system to form secondary tumors in distant organs [142]. The tumor microenvironment (TME) plays a pivotal role in regulating the aggressiveness, progression, and dissemination of malignant cells [27,143]. Therefore, accurately replicating the extracellular matrix (ECM) of the TME is essential for developing effective BC models.

Simulated microgravity was employed in this study to investigate how varying ECM stiffness influences tumor aggressiveness and progression. Different ECM stiffness levels were created using GelMA and PEGDA hydrogels: 3 % GelMA/3 % PEGDA (3 %), 5 % GelMA/3 % PEGDA (5 %), and 7 % GelMA/3 % PEGDA (7 %). In this study, the various types ECM stiffness were cultured in simulated microgravity and protein and gene expression were investigated to understand the effect of the gravity vector or lack thereof on the BC cells.

First, the printability of the 96 well-plate adapted platform, a new technological addition to the bioprinter, was tested. The bioprinted objects were evaluated for consistency across each condition, and while printability decreased with increasing GelMA concentration, the differences between conditions were not significant. Then, cell viability and ATP production were assessed. Results demonstrated that cells thrived better in a stiffer microenvironment (7 %), highlighting the importance of ECM stiffness in modeling the BC microenvironment. Lower stiffness levels did not adequately represent a thriving tumor environment, which confirmed previous tumor rheology measurements.

Cell proliferation, assessed using the marker Ki67, did not show significant differences between simulated microgravity (0g) and normal gravity (1g) conditions. However, Yes-associated protein (YAP), a mechanotransducer that responds to ECM rigidity by relocalizing in the nucleus in stiffer environment [144] and is associated with tumor growth and metastasis to drives progression of the tumor in BC [[95], [96], [97]], showed a distinct behavior. YAP was increasingly localized to the nucleus in simulated gravity (0g) in printed constructs – while YAP localized to the cytoplasm in controls (non-printed spheroids). The leading “buds” of the spheroids in printed constructs seemed to have a particularly intense YAP signal (see 5 %0g and 7 %0g). In earth gravity (1g), YAP was localized to the cytoplasm, in printed spheroids as in the controls, proving that, for this condition, the matrix had no influence on YAP. A previously cited study investigated the effect of YAP on colorectal cancer cells in simulated microgravity and found similar results (0g led to the nuclear localization of YAP) using single cells [91]. This suggests that microgravity may corrupt the effects of ECM stiffness on YAP localization.

Further analysis of Epithelial Cell Adhesion Molecule (EpCAM) and Vimentin indicated confirmed the previous results in MCF-7 cells: EMT was observed in 0g at higher stiffness (5 % and 7 %) whereas it was faintly present at 1g. As MCF-7 has been shown to naturally overexpress vimentin, a second cell line, MDA-MB-231 (triple negative), was also used, showing clear epithelial-to-mesenchymal transition (EMT) markers at higher stiffness under 1g conditions, which were less pronounced at 0g. In this aggressive cell line, we could see a clear EMT triggered on one hand by the matrix stiffness (7 %1g) and on the other hand by overall lack of ECM (control 1g), effects that were less prominent or entirely negated in 0g. RNA sequencing results confirmed these findings. A recent study from Filiz, Arslan et al. [107] showed similar results, even though the MCF-7 cells were only subjected to microgravity during spheroid formation.

The size of the nuclei as well as the ratio cytoplasm to nucleus (karyoplasmic ratio) were investigated and we showed that the softer bioprints generally had a larger nucleus in term of volume than the control, regardless of the gravity condition, but the volume was higher at 7 %1g and not 7 %0g which indicated a negative effect of microgravity on the nuclear size. The karyoplasmic volume indicated that the softer hydrogel had less cytoplasm compared to the 7 % and control in 0g, but the 1g conditions were homogenous and had generally bigger ratios, an effect reminiscing of the low cell viability previously demonstrated. There was no significant difference noted between the controls in 1g and 0g. The high variance observed in softer conditions was more pronounced under 1g than under 0g, demonstrating that simulated microgravity mitigated nuclear size deviations. We conclude from these results that the presence of matrix affected the morphology of the cell in 0g compared to 1g in a very stiff environment, meaning that the aggressivity of BC linked to the highly stiff EMC was reversed in 0g. A similar effect, namely a decreased cell area has been measured in osteosarcoma cells exposed to microgravity [145,146] and linked to a decrease in maturation of focal adhesion spots in microgravity.

RNA sequencing and differential gene expression analysis revealed that the bioprinted constructs at 1g and 0g never displayed similar gene expression when compared to control 1g, indicating clear reversed patterns. Bioprinted constructs in 1g exhibited a lower disposition for aggressiveness and proliferation compared to 0g conditions. There were additionally linked to apoptosis and NF-kB pathway regulation, indicating that microgravity suppressed cell death in BC cells. It has been shown in simulated microgravity studies in BC that the NF-kB pathway was activated in cells forming 3D structures from single floating cells [147,148], proving that the process of spheroid formation worked differently than when the spheroids were preformed and encapsulated in an ECM, the latter being more representative of *in vivo* conditions. Genes upregulated in simulated microgravity but downregulated at 1g were linked to chemoresistance and poor prognostic. The expression of prognostic markers in 7 %1g and control 0g could be reversed in 7 %0g, suggesting that the impact of simulated microgravity surpassed that of ECM stiffness for those genes. Taken together, those results clearly showed that the effect of the ECM was reversed in simulated microgravity.

Molecular pathways were additionally identified, conditions in simulated microgravity (7 %0g and control 0g) showed an enrichment in the NADH dehydrogenase complex, linked to BC aggressivity and autophagy. Autophagy, in the context of ubiquitination, was more prominent in 1g conditions (7 %1g vs. control 1g), whereas cadherin binding and EMT were significant in 0g conditions (7 %0g vs. control 0g). Comparing bioprinting conditions (7 %0g vs. 7 %1g) highlighted the importance of both cadherin binding and the ubiquitin-proteasomal complex. This confirmed that microgravity promotes cell death through apoptosis regulation, and that EMT and autophagy are critical processes for bioprinted constructs under simulated microgravity compared to 1g conditions, increasing cell-matrix interactions and influencing metastasis. These findings are consistent with the increased nuclear localization of YAP and the bigger karyoplasmic measurements in bioprinted constructs at 0g. The lack of EMT in 0g was also consistent with the EpCAM and vimentin results.

## 5 Conclusion

In conclusion, the application of simulated microgravity to BC cells can significantly influence the effects of the ECM, potentially reversing the impact of a stiffer tumor microenvironment. Our results sometimes conflict with previous studies conducted during the spheroid formation, hinting at the crucial role the ECM plays. Thanks to the use of microgravity, a dimension was added to this study that is seldom considered in drug discovery. Our approach offers valuable insights into the underlying molecular mechanisms and could identify novel therapeutic targets. For instance, MAM Domain Containing Glycosylphosphatidylinositol Anchor 2 (MDGA2), EF-Hand Calcium Binding Domain 10 (EFCAB10), MT-RNR2-Like 3 and 6 (MTRNR2L3/6), Ribonucleic Acid Export 1 (RAE1) and Translin Associated Factor X Interacting Protein 1 (TSNAXIP1), which have not been studied extensively or at all as prognostic or therapeutic targets in BC, emerged as notable candidates in this study and warrant further investigation. Utilizing simulated microgravity in cancer research could thus uncover previously unexplored pathways and markers, contributing to the development of more effective treatments.

## CRediT authorship contribution statement

**Louise Breideband:** Writing – review & editing, Writing – original draft, Visualization, Investigation, Formal analysis, Data curation, Conceptualization. **Kaja Nicole Wächtershäuser:** Writing – review & editing, Visualization, Investigation. **Ryan Sarkar:** Writing – review & editing, Methodology, Investigation. **Melosha Puspathasan:** Writing – review & editing, Methodology, Investigation. **Ernst H.K. Stelzer:** Writing – review & editing, Resources, Project administration. **Francesco Pampaloni:** Writing – review & editing, Writing – original draft, Supervision, Resources, Project administration, Conceptualization.

## Declaration of generative AI and AI-assisted technologies in the writing process

During the preparation of this work the author(s) used ChatGPT in order to improve legibility and language. After using this tool/service, the author(s) reviewed and edited the content as needed and take(s) full responsibility for the content of the published article.

## Declaration of competing interest

The authors declare the following financial interests/personal relationships which may be considered as potential competing interests: Francesco Pampaloni reports financial support was provided by European Union. If there are other authors, they declare that they have no known competing financial interests or personal relationships that could have appeared to influence the work reported in this paper.

## Data Availability

Data will be made available on request.
