# Supplementary material for: Gravitational forces and matrix stiffness modulate the invasiveness of breast cancer cells in bioprinted spheroids
Source: Mater Today Bio. 2025 Mar 4;31:101640. doi: 10.1016/j.mtbio.2025.101640 (PMC11930500; doi:10.1016/j.mtbio.2025.101640)
Supplement: Multimedia component 1 [file mmc1.docx]

Supplementary Material Breideband et al. 2024

“Gravitational Forces and Matrix Stiffness Modulate the Invasiveness of Breast Cancer Cells in Bioprinted Spheroids”


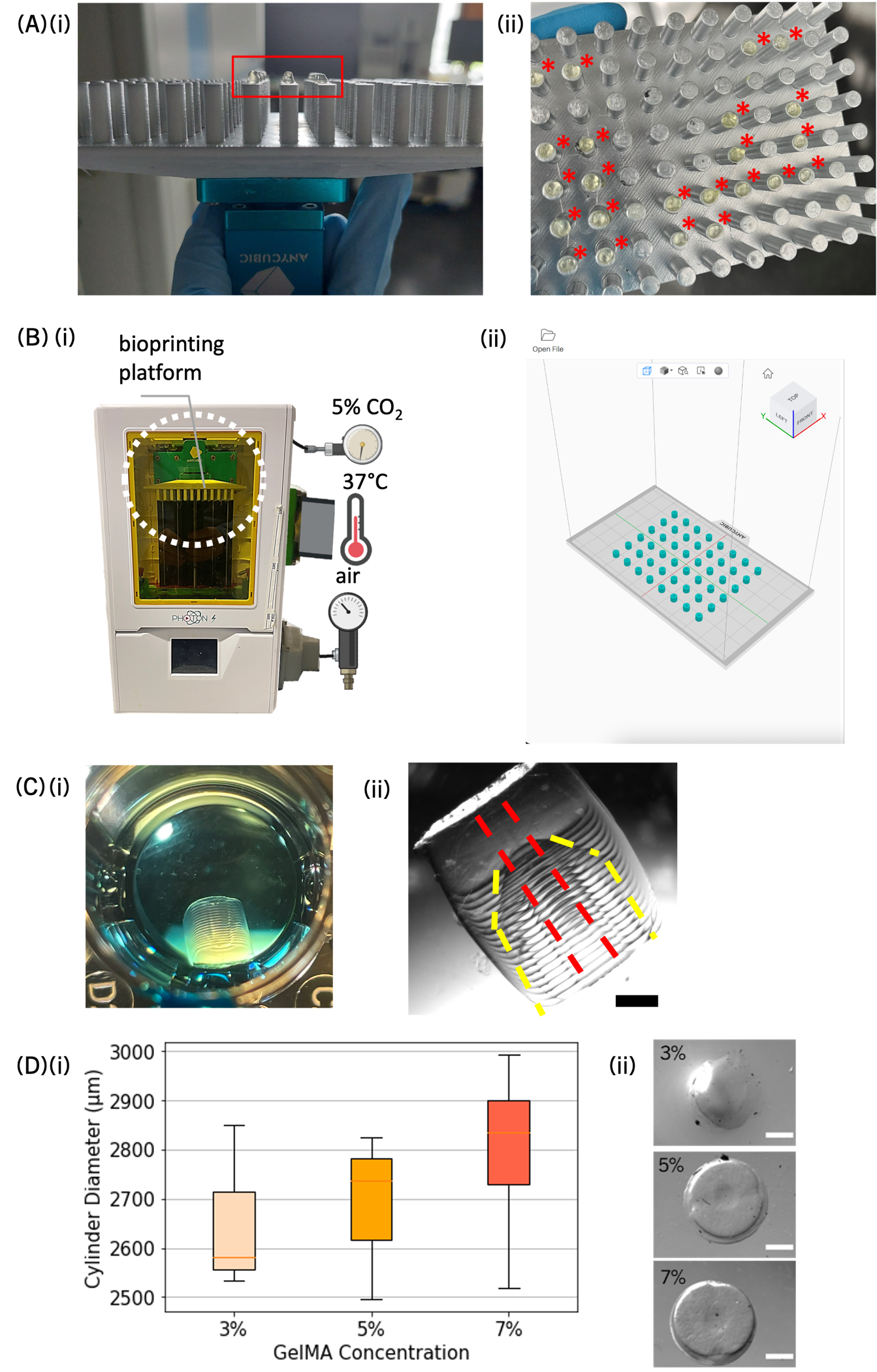


**Supplementary Fig. 1** – Test printing of branched channels on the custom-made 96 well plate platform. (A) Overview of the bioprinted cylinders directly after printing, from the side (i) and from the top (ii). The bioprinted objects are highlighted with a red rectangle (i) or with a red asterisk (ii). (B) Adapted 3D bioprinter can print 96 constructs at once. (i) Picture of the machine with the 96 platforms and temperature and CO_2_ controls, with pressurized air to cool down the LED array. (ii) CAD model displayed in the slicing software of 48 cylinders distributed to fit in a 96 well-plate. (C) Visualization of the cylinders used for the bioprinting. (i) Image of the construct inside a well of a 96 well-plate. (ii) Images of the cylinders with the resulting channels highlighted with red (main channel) and yellow (branching side channels) lines. (D) Microscope: Zeiss SteREO Discovery V8. Objective: Plan Apo S, 0.63x FWD 81 mm. Camera: AxioCam IcC SIN. Pixel size: 4.54 x 4.54 µm². Scale bar: 500 µm.


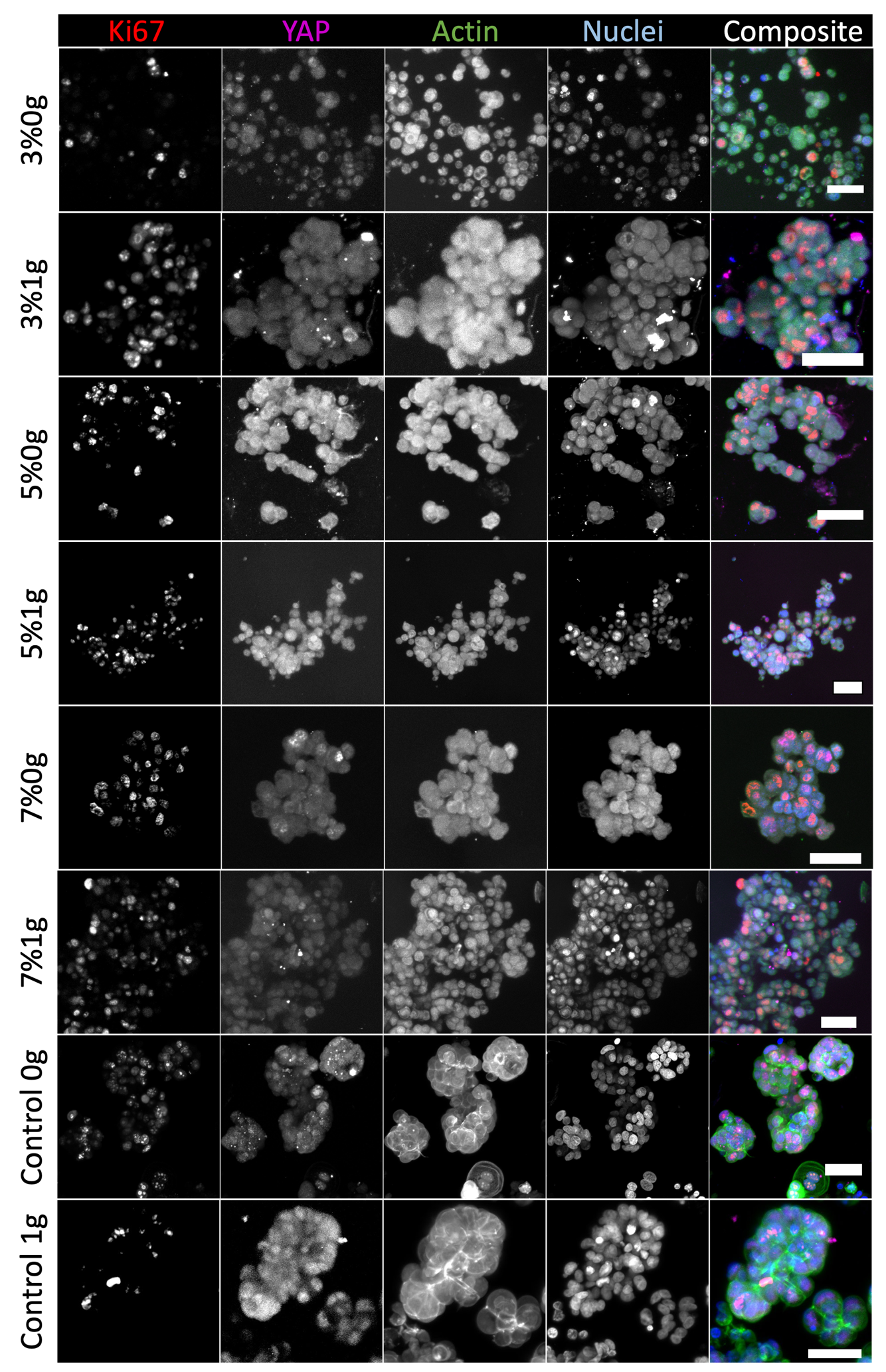


**Supplementary Fig. 2** Representative pictures of immunofluorescent staining of Ki67 (proliferation marker, in red) and Hoechst (nuclei, in cyan) for samples bioprinted in matrices of increasing stiffnesses (3%, 5% and 7%) and for non-printed spheroid controls, cultured in simulated microgravity (0g) and normal gravity (1g). Data shown is from one experiment representative of three biological replicates and three technical replicates. The samples were stained against Ki67 in red, YAP in magenta, Phalloidin for the actin cytoskeleton in green and Hoechst 34580 for the nuclei in blue. Microscope: Zeiss AxioObserver LSM780. Objective: Plan ApoChromat 20×/0.8 M27. Scale bars=50 μm.


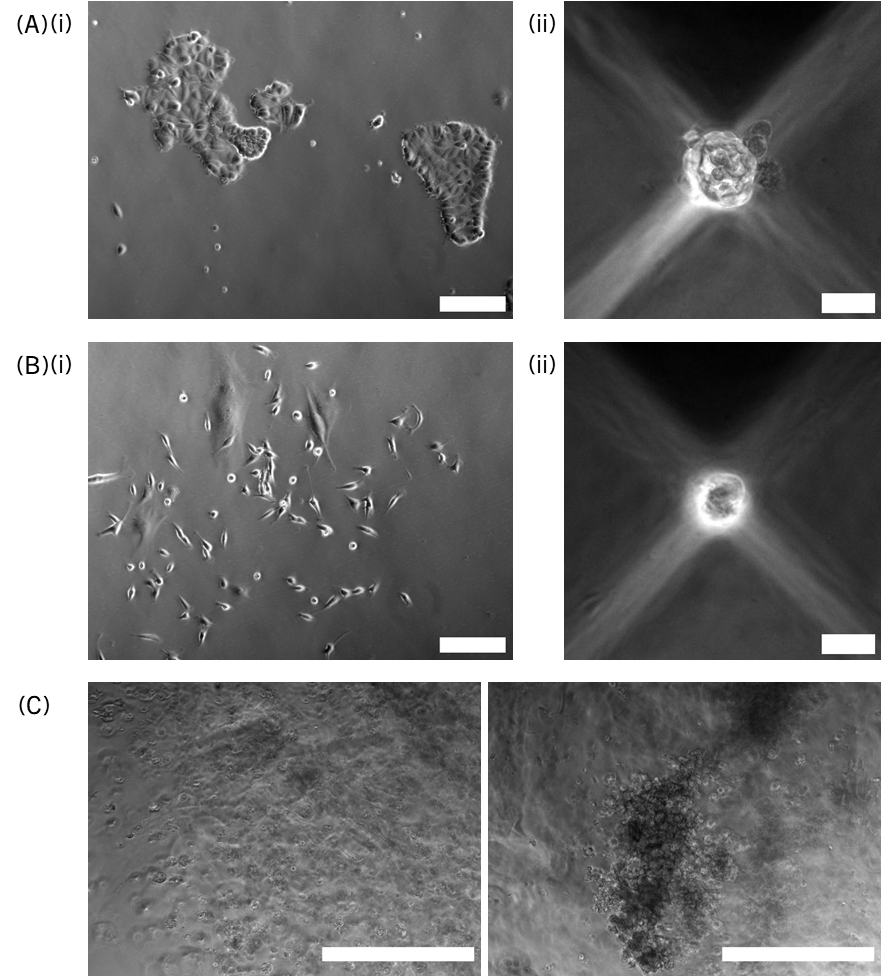


**Supplementary Fig. 3** – Microscopic observation of the breast cancer cells cultured in two and three dimensions. (A) Phase contrast imaging of the MCF-7 cells cultured in a flask (scale bar: 200 µm) (i) and in a Sphericalplate 5D (scale bar: 50 µm) (ii). (B) Phase contrast imaging of the MDA-MB-231 cells cultured in a flask (scale bar: 200 µm) (i) and in a Sphericalplate 5D (scale bar: 50 µm) (ii). (C) Phase contrast images of MCF-7 spheroids bioprinted in a 7% hydrogel (scale bar: 500 µm). Microscope: Zeiss SteREO Discovery V8. Objective: Plan Apo S, 0.63×/0.116 FWD 81 mm. Camera: AxioCam ICm1 S/N 285852050. Pixel size: 0.93 × 0.93 µm2.


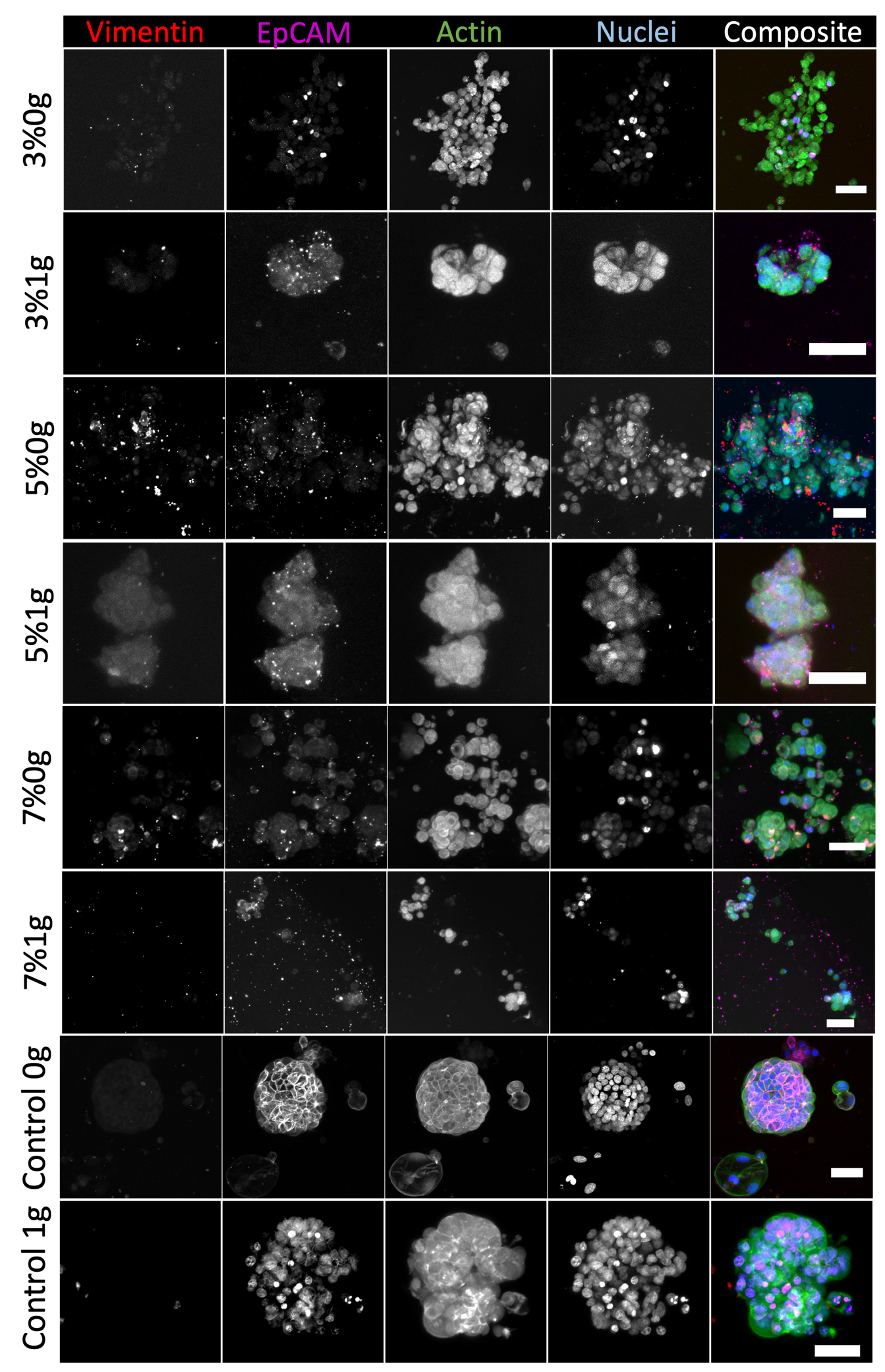


**Supplementary Fig. 4** – Immunofluorescent staining of MCF-7 cells showing vimentin, EpCAM, phalloidin (actin) and Hoechst 34580 (nuclei) for samples bioprinted in matrices of increasing stiffnesses (3%, 5% and 7%) and for non-printed spheroid controls, cultured in simulated microgravity (0g) and normal gravity (1g). Data shown is from one experiment representative of three biological replicates and three technical replicates. Microscope: Zeiss AxioObserver LSM780. Objective: Plan ApoChromat 20×/0.8 M27. Scale bars=50 μm.


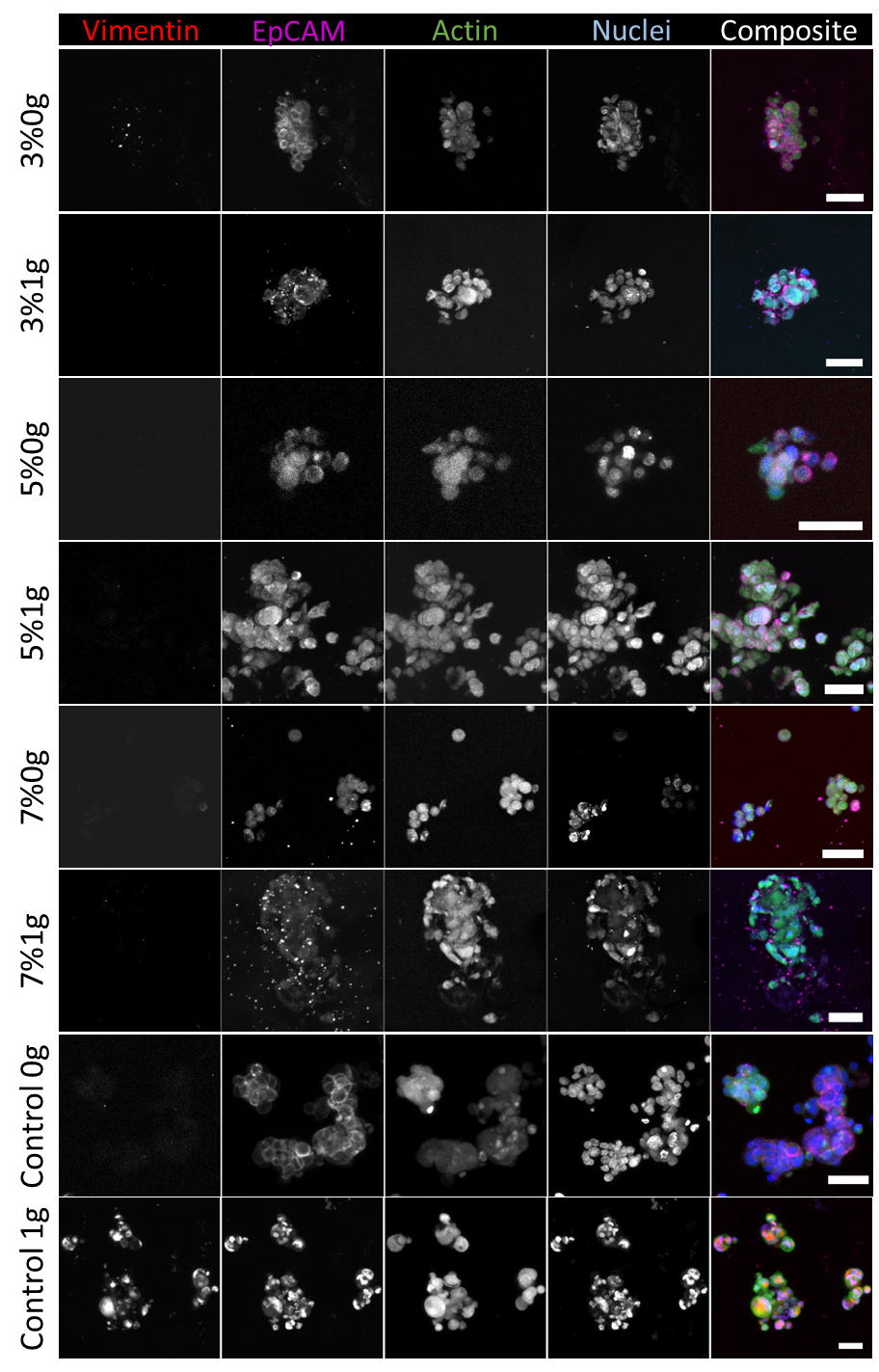


**Supplementary Fig. 5** – Immunofluorescent staining of MDA-MB-231 cells showing vimentin, EpCAM, phalloidin (actin) and Hoechst 34580 (nuclei) for samples bioprinted in matrices of increasing stiffnesses (3%, 5% and 7%) and for non-printed spheroid controls, cultured in simulated microgravity (0g) and normal gravity (1g). Data shown is from one experiment representative of three biological replicates and three technical replicates. Microscope: Zeiss AxioObserver LSM780. Objective: Plan ApoChromat 20×/0.8 M27. Scale bars=50 μm


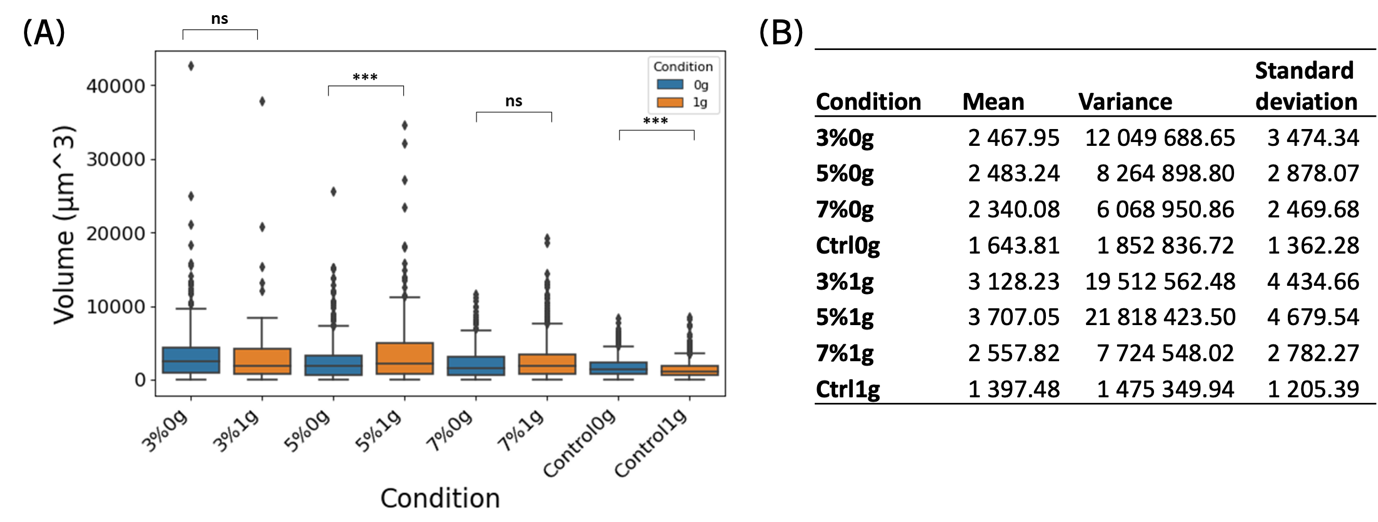


**Supplementary Fig. 6** - Assessment of the nuclei volumes for all conditions. (A) Plot of the nuclei volumes for samples grown in simulated microgravity (0g, left) and normal gravity (1g, right). The samples showed a significant decrease compared to their respective controls (3%1g – 3%1g: p=-18, 5%0g: 4.03e-4, 3%1g: p=6.71e-8, 5%1g: p=2.60e-18, 7%1g: p=4.76e-13). The samples were tested for normality with a Kolmogorov-Smirnov test. The data is tested for significance with a Mann-Whitney U-test, n = 128 to 704, p < 0.005 (***), p < 0.01 (**) and p < 0.05 (*). (B) Mean-variance table for the nuclei volumes (in µm3).


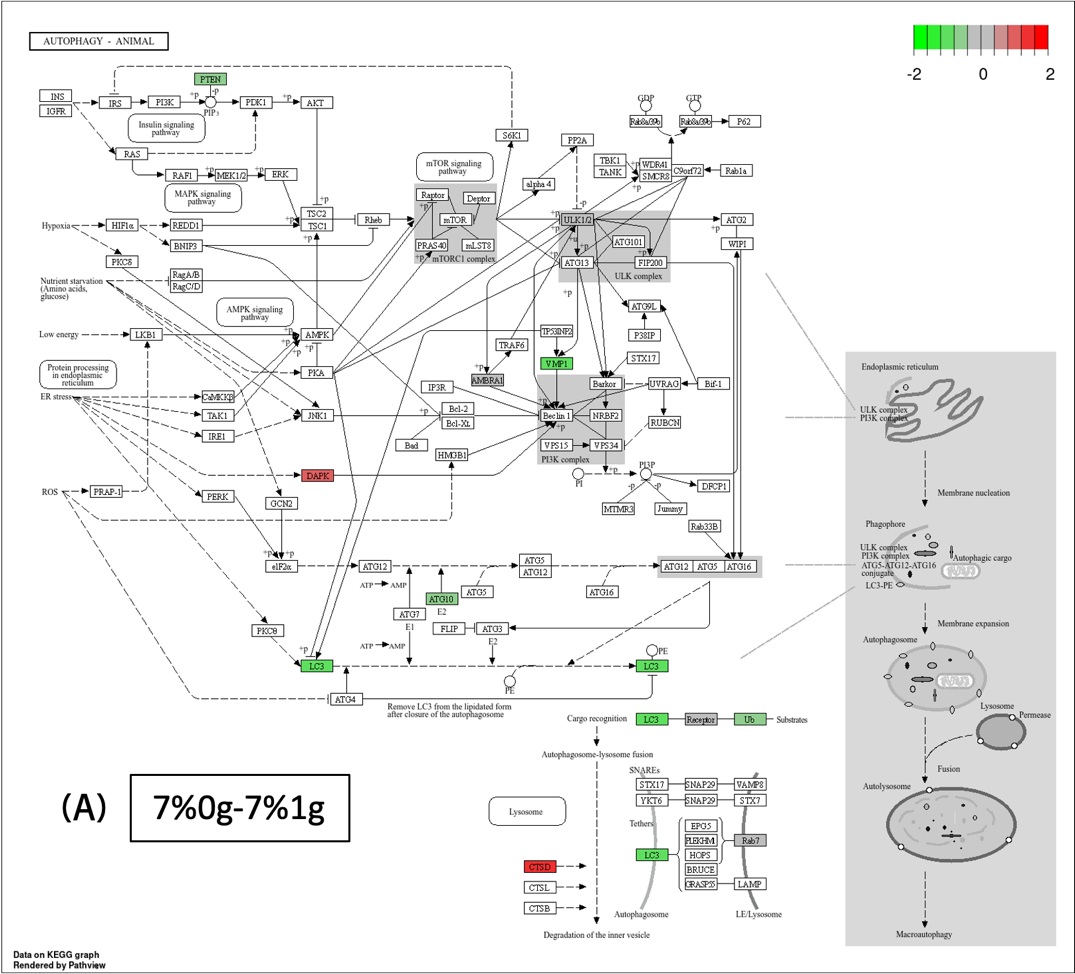

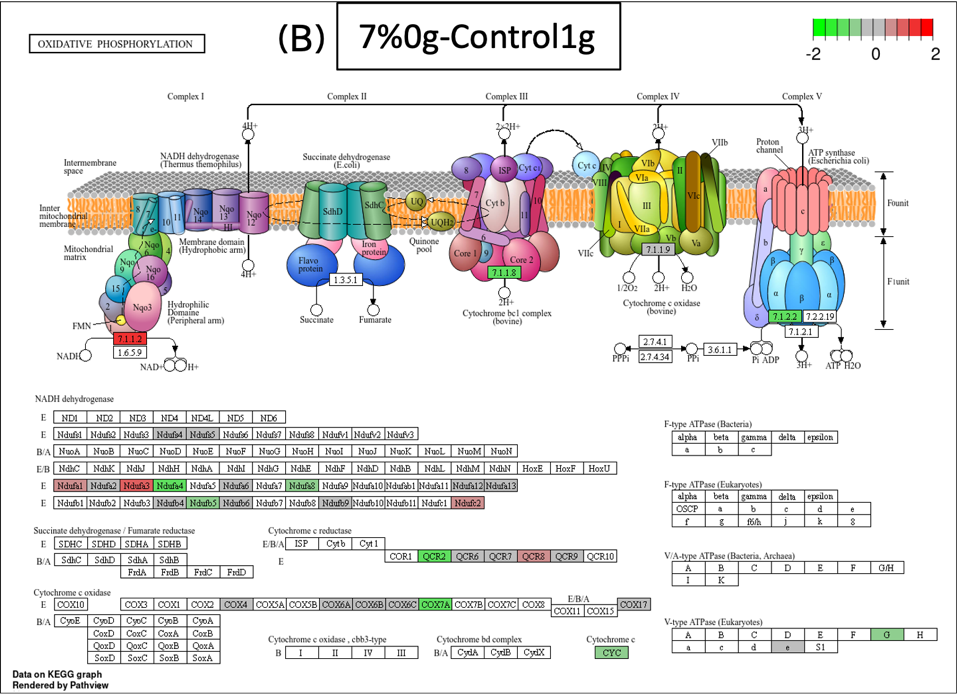


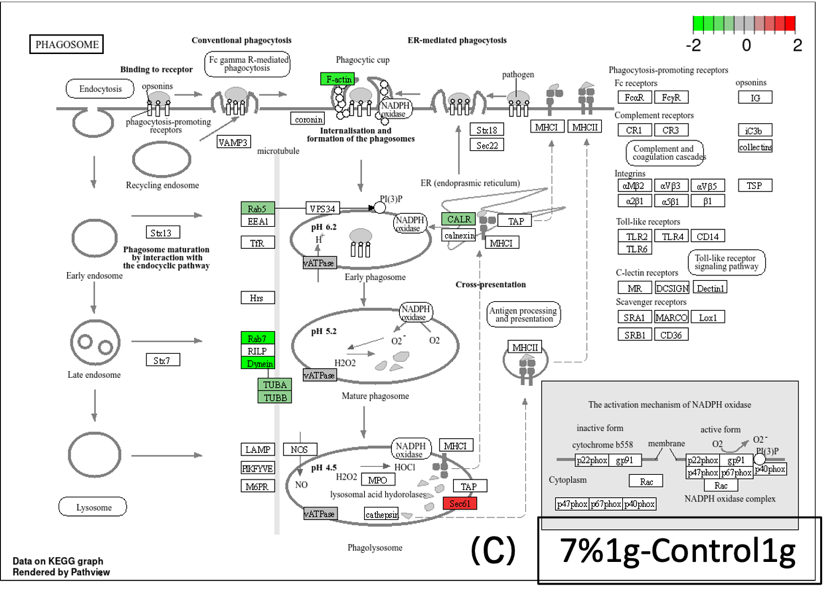

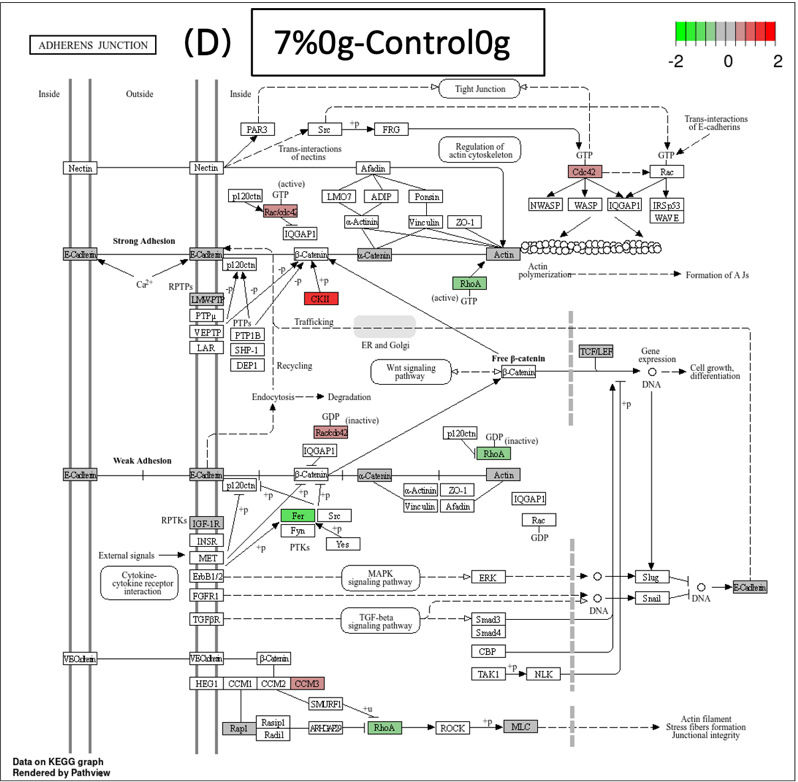


**Supplementary Fig. 7** – Molecular Function of over- or underexpressed genes for the different conditions as part of KEGG (Kyoto Encyclopedia of Genes and Genomes) pathways. Produced using SR plots.

| **Antibody** | **Supplier** | **Cat. Number** | **Host** | **Dilution** | **Secondary Antibody** | **Dilution** |
| --- | --- | --- | --- | --- | --- | --- |
| Ki67 | Cell Signalling | 8D5 | Mouse | 1:400 | Donkey anti-mouse 568 | 1:400 |
| YAP | Cell Signalling | D8H1X | Rabbit | 1:100 | Goat anti-rabbit 488 | 1:400 |
| EpCAM | Cell Signalling | VU1D9 | Mouse | 1:100 | Donkey anti-mouse 568 | 1:400 |
| Vimentin | Cell Signalling | D21H3 | Rabbit | 1:100 | Goat anti-rabbit 488 | 1:400 |

**Supplementary Table 1** – List of primary and secondary antibodies

| Dye | Supplier | Cat. Number | Dilution |
| --- | --- | --- | --- |
| Propidium Iodide | Sigma Aldrich | P4170-10MG | 1:100 |
| Fluorescein Diacetate | Sigma Aldrich | F7378-5G | 1:500 |
| Hoechst 33342 | ThermoFisher Scientific | H1399 | 1:500 |
| iFluor Phalloidin 647 | BioMol | ABD-23127 | 1:200 |
| Hoechst 34580 | ThermoFisher Scientific | H21486 | 1:400 |

**Supplementary Table 2** – List of dyes
